# Activin-A impairs CD8 T cell-mediated immunity and immune checkpoint therapy response in melanoma

**DOI:** 10.1136/jitc-2022-004533

**Published:** 2022-05-16

**Authors:** Katarina Pinjusic, Olivier Andreas Dubey, Olga Egorova, Sina Nassiri, Etienne Meylan, Julien Faget, Daniel Beat Constam

**Affiliations:** 1 School of Life Sciences (SV), ISREC, Ecole Polytechnique Federale de Lausanne, Lausanne, Switzerland; 2 Bioinformatics Core Facility, Swiss Institute of Bioinformatics, Lausanne, Switzerland; 3 Laboratory of Immuno-Oncology, Bordet Cancer Research Laboratories, Institut Jules Bordet, Faculty of Medicine, and Laboratory of Immunobiology, Faculty of Sciences, Université Libre de Bruxelles, Bruxelles, Belgium; 4 Equipe Immunity and Cancer IRCM, INSERM U1194, Montpellier, France

**Keywords:** melanoma, immunotherapy, immune evasion, tumor microenvironment

## Abstract

**Background:**

Activin-A, a transforming growth factor β family member, is secreted by many cancer types and is often associated with poor disease prognosis. Previous studies have shown that Activin-A expression can promote cancer progression and reduce the intratumoral frequency of cytotoxic T cells. However, the underlying mechanisms and the significance of Activin-A expression for cancer therapies are unclear.

**Methods:**

We analyzed the expression of the Activin-A encoding gene *INHBA* in melanoma patients and the influence of its gain- or loss-of-function on the immune infiltration and growth of *BRAF*-driven YUMM3.3 and iBIP2 mouse melanoma grafts and in B16 models. Using antibody depletion strategies, we investigated the dependence of Activin-A tumor-promoting effect on different immune cells. Immune-regulatory effects of Activin-A were further characterized in vitro and by an adoptive transfer of T cells. Finally, we assessed *INHBA* expression in melanoma patients who received immune checkpoint therapy and tested whether it impairs the response in preclinical models.

**Results:**

We show that Activin-A secretion by melanoma cells inhibits adaptive antitumor immunity irrespective of *BRAF* status by inhibiting CD8^+^ T cell infiltration indirectly and even independently of CD4 T cells, at least in part by attenuating the production of CXCL9/10 by myeloid cells. In addition, we show that Activin-A/*INHBA* expression correlates with anti-PD1 therapy resistance in melanoma patients and impairs the response to dual anti-cytotoxic T-Lymphocyte associated protein 4/anti-PD1 treatment in preclinical models.

**Conclusions:**

Our findings suggest that strategies interfering with Activin-A induced immune-regulation offer new therapeutic opportunities to overcome CD8 T cell exclusion and immunotherapy resistance.

**Key messages**

- Expression of the TGFβ family member Activin-A encoded by the *INHBA* gene correlates with poor prognosis in many cancer types. Secretion of Activin-A by melanoma cells promotes tumor growth and correlates with reduced CD8^+^ T cell infiltration, but underlying mechanisms and their relevance for immunotherapies were unknown. This study shows that Activin-A indirectly dampens CD8^+^ T cells responses in melanoma, at least in part by directly impairing CXCL9 and CXCL10 secretion by myeloid cells in the tumor, leading to resistance to the immune checkpoint blockade therapy by combined anti-PD1/anti-cytotoxic T-Lymphocyte associated protein 4 treatment. These findings identify Activin-A as a promising new target to overcome immunotherapy resistance in melanoma and possibly other cancers.

## Introduction

Tumor immune surveillance by cytotoxic T lymphocytes (CTLs) and natural killer (NK) cells is frequently compromised by co-inhibitory receptors such as cytotoxic T-lymphocyte associated protein (CTLA-4) and programmed cell death 1 (PD1) that promote CTL exhaustion and immune self-tolerance.^1 2^ Thus, in cancers that are chronically inflamed by a high mutational burden, therapeutic anti-PD1 and anti-CTLA4 antibodies frequently reinvigorate antitumor immunity and drastically improve progression-free survival.^3^ In melanoma, therapy responsiveness also correlates with an IFNγ gene expression signature that includes antigen-presenting major histocompatibility complex (MHC) class I proteins.^4^ Conversely, low mutational burden, decreased antigen presentation, inhibition of CTL priming or infiltration, and their premature exhaustion all contribute to therapy resistance.^5^

Activins and Inhibins are gonadal hormones regulating the release of follicle-stimulating hormone. Inhibin antagonism of Activin-A signaling also protects against gonadal tumors and cancer-associated muscle wasting (cachexia).^6^ Activin-A encoded by the *INHBA* gene binds type II activin receptors (ACVR2) and activin receptor-like kinase 4 (ALK4, also known as ACVR1B) to activate SMAD2 and SMAD3 transcription factors. By contrast, receptor complexes containing ALK7 (ACVR1C) preferentially bind Activin-B and Activin-AB.^7^ While downregulation of Activin receptor signaling within cancer cells increases their proliferation and tumor progression in pituitary, pancreas, esophagus, and colon,^8–11^ aberrant ALK4 signaling can promote cancer cell invasiveness, metastasis, and resistance to chemotherapy.^12 13^ *INHBA* mRNA is upregulated across diverse tumor types, including various skin malignancies,^14 15^ and elevated circulating Activin-A promotes cachexia and correlates with poor prognosis.^16^ However, whether Activin-A promotes or inhibits tumor progression depends on context.^14^ A dual role has been reported in melanoma, where ALK4 signaling in melanocytes inhibits proliferation and survival, whereas frequent gain of Activin-A secretion by melanoma cells promotes tumor growth and metastasis by inhibiting adaptive antitumor immunity.^15 17^ In addition, Activin-A increases tumor angiogenesis,^15^ possibly by enriching proangiogenic tumor-associated macrophages (TAMs) in the tumor microenvironment (TME), as demonstrated in skin squamous cell carcinoma.^18^ Activin-A is also secreted by macrophages themselves, at least in vitro.^19–21^ In addition, autocrine Activin-A signaling dampens proinflammatory cytokine and chemokine release by cultured blood monocytes and monocytic dendritic cells (DCs).^22 23^ Cultured CD4^+^ T cells express Activin-A after T cell receptor stimulation, specifically in Th2 and less in Th1 subsets.^19^ By contrast, Tregs can acquire *Alk7* expression and signaling during TGFβ-induced differentiation, and Activin-neutralizing antibodies were reported to reduce Treg frequency in syngeneic B16 melanoma grafts.^24^ Here, we compare the impact of Activin-A on immune infiltrates in B16-F1 versus BRAF-driven mouse melanoma and test its effect on the response to immunotherapy. We show that melanoma cell-derived Activin-A indirectly inhibits CTL accumulation and proliferation, likely by interfering with the CXCL9/10-CXCR3 chemokine axis, and that its expression correlates with resistance to anti-PD1 therapy in melanoma patients and impairs the response to an anti-PD1/anti-CTLA4 combination in BRAF-driven mouse melanoma. Thus, inhibition of Activin-A emerges as a promising strategy to interfere with tumor-induced immune evasion and immunotherapy resistance.

## Materials and methods

The complete experimental procedures and protocols are described in online supplemental material.

10.1136/jitc-2022-004533.supp1 Supplementary data

## Results

### Activin-A secretion by melanoma cells decreases CTL and NK cell infiltration

**Figure 1:**
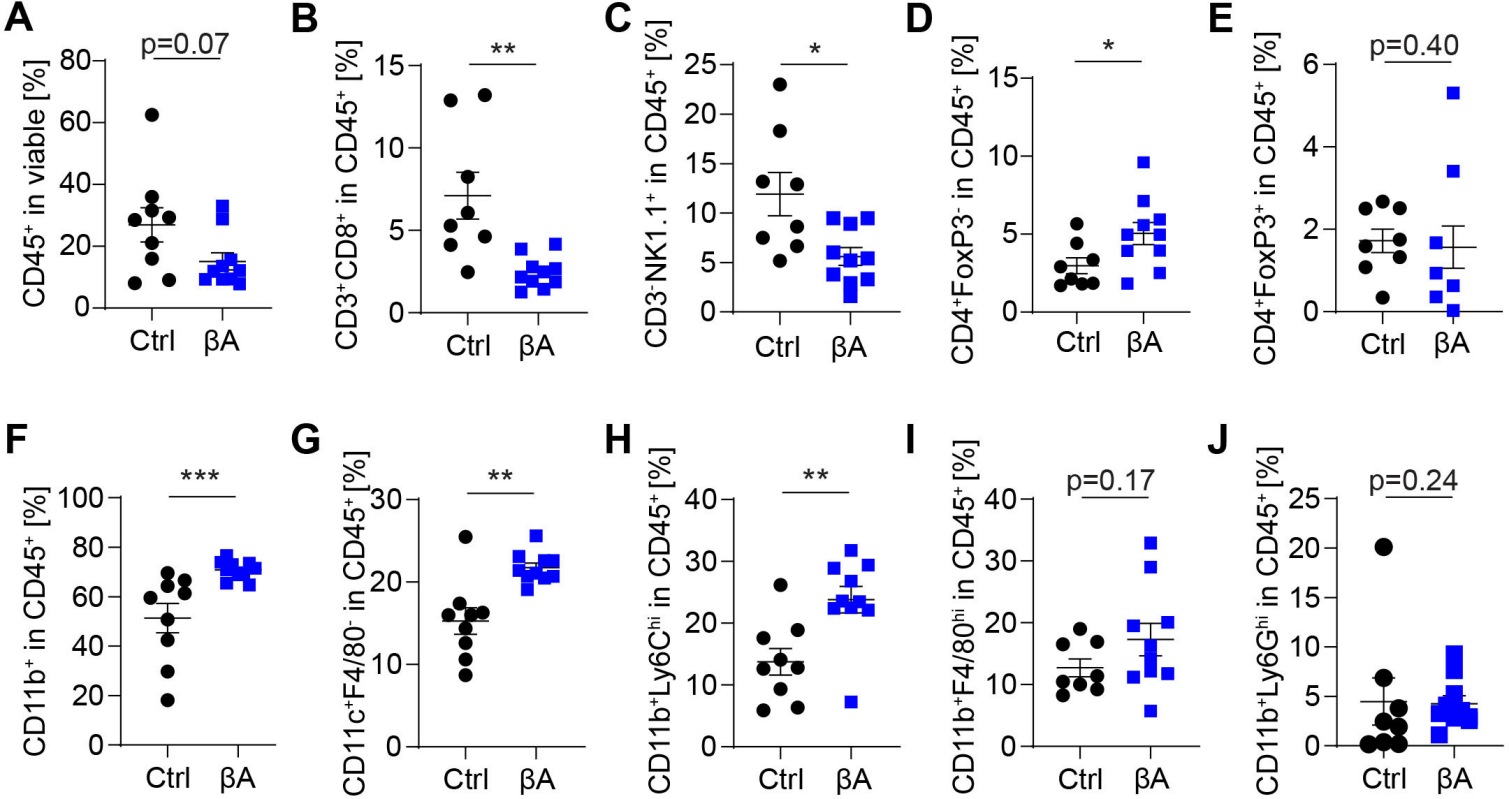
B16-F1 melanoma cell-derived Activin-A decreases tumor-infiltrating CTLs and NK cells. (A) Percentages of total CD45^+^ immune cells, and (B) CD3^+^CD8^+^ T cells, (C) CD3^-^NK1.1^+^ NK cells, (D) CD4^+^FoxP3^-^ T cells, (E) CD4^+^FoxP3^+^ Tregs, (F) CD11b^+^ myeloid cells, (G) CD11c^+^F4/80^-^ dendritic cells, (H) CD11b^+^Ly6C^hi^ monocytes, (I) CD11b^+^F4/80^hi^ TAMs, and (J) CD11b^+^Ly6G^hi^ neutrophils among CD45^+^ immune cells in syngeneic B16F1-Ctrl (n=8) and B16F1-βA (n=10) mouse melanoma grafts, quantified at the endpoint by flow cytometry. Data represent SEM, *p<0.05, **p<0.01, ***p<0.001, Student’s t-test. CTLs, cytotoxic T lymphocytes; NK, natural killer.

Overexpression of Activin-A in B16-F1 mouse melanoma cells that do not transcribe endogenous *INHBA* can promote tumor immune evasion and metastatic growth in syngeneic hosts.^15^ To further validate this preclinical model, we first measured circulating Activin-A levels in the plasma of mice with B16-F1 mouse melanoma grafts expressing lentiviral *INHBA* (B16F1-βA) or empty vector control (B16F1-Ctrl). Analysis by ELISA at the endpoint revealed that gain of *INHBA* expression increased the average plasma level of Activin-A from 0.9 to 34.7 ng/mL (p=0.0015, online supplemental figure S1A). By comparison, concentrations between 59 to 215 ng/mL have been reported in plasma of granulosa-cell-derived tumors in female Inhibin-deficient mice that secrete endogenous Activin-A.^25^ As a first crude estimate of the net effect on proinflammatory and anti-inflammatory signals, we considered whether Activin-A altered the expression of specific effectors of tumor cell killing. Analysis of total RNA showed that *INHBA*-expressing tumors transcribed lower levels of *Gzmb, Prfn1,* and *Il1b* (online supplemental figure S1B–D), even though regulatory factors such as *Il2* and *Il10* were unchanged, at least at the mRNA level, and trends for decreased expression of *Ifng* and *Tnfa* mRNAs did not reach significance (online supplemental figure S1E–H). To survey the immune landscape of B16F1-βA and -Ctrl melanoma grafts in an unbiased manner, we analyzed tumor-infiltrating leucocytes (TILs) by flow cytometry using a 16-color antibody panel (online supplemental table S1). We observed a clear trend of Activin-A to diminish immune infiltrates that was associated with a significant reduction of CTL and NK cell proportions (figure 1A–C). Conversely, the proportion of conventional CD4 T cells increased, although without altering the abundance of CD4^+^ FoxP3^+^ regulatory T cells (figure 1D, E). Melanoma cell-derived Activin-A also enriched CD11b^+^ myeloid cells, CD11b^+^CD11c^+^F4/80^-^ DC, as well as CD11b^+^Ly6C^hi^ monocytes (figure 1F–H). By contrast, F4/80^hi^ TAMs that were highly abundant, and neutrophils gated as CD11b^+^Ly6G^hi^ immune population, and rare B cells did not change in frequency in B16F1-βA compared with control tumors (figure 1I, J and online supplemental figure S1I). To assess whether Activin-A influenced immune cell proliferation, we examined Ki67 expression. The fraction of proliferating CTLs decreased by 23% in B16F1-βA compared with B16F1-Ctrl tumors (online supplemental figure S1J). By contrast, no change was seen in the proliferation of tumor-infiltrating NK cells or Tregs, and the proliferation of FoxP3^-^ CD4^+^ T cells increased in βA-secreting tumors (online supplemental figure S1K–M). Together, these results show that Activin-A secretion by melanoma cells shifts the composition of tumor immune infiltrates from CTLs and NK cells toward increased non-regulatory CD4 T cells, DCs, and monocytes (online supplemental figure S1N).

### Activin-A secretion by B16.OVA melanoma stimulates intratumoral antigen presentation

**Figure 2:**
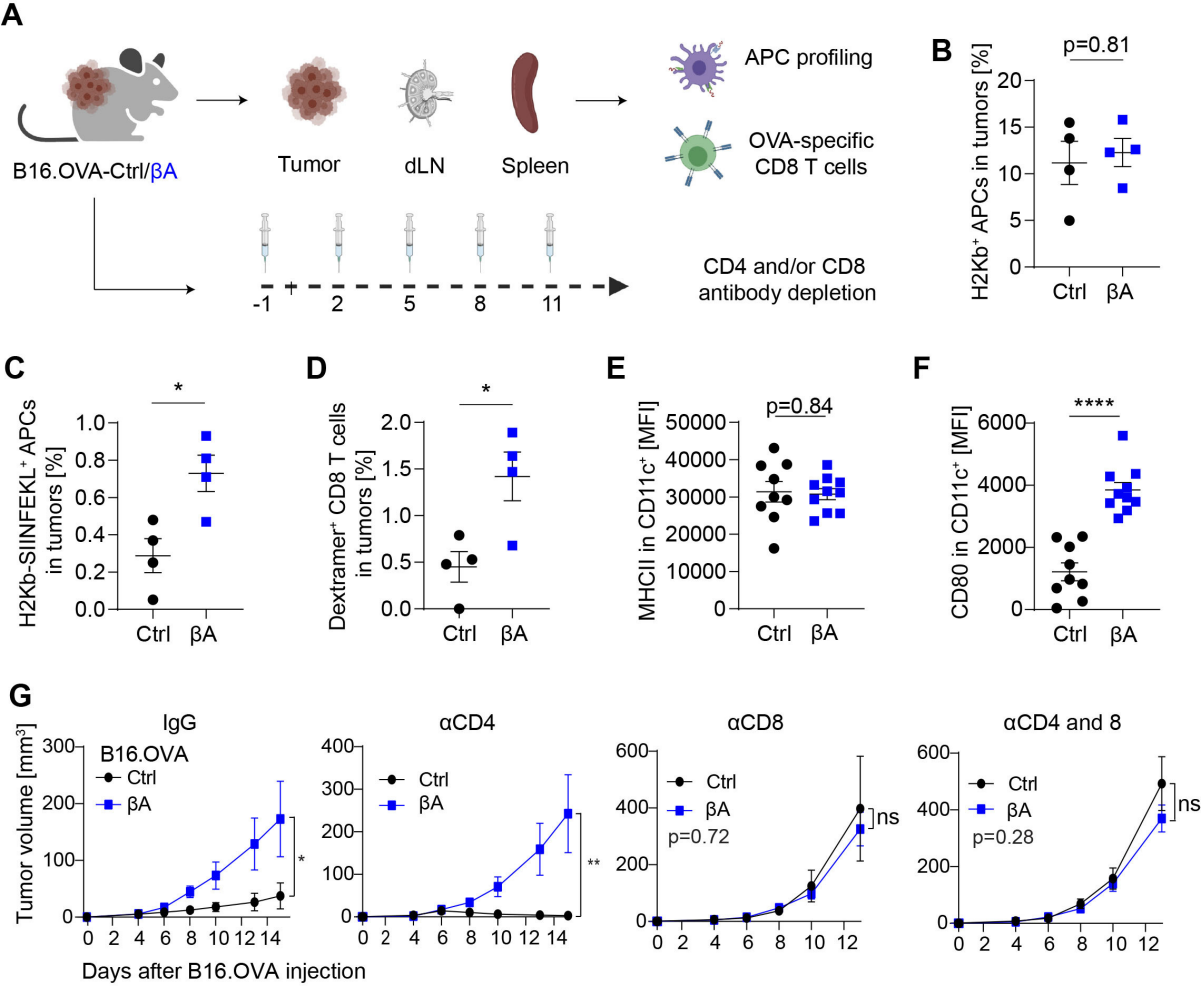
Activin-A accelerates melanoma growth by inhibiting antigen-specific CTLs, independently of CD4^+^ T cells and despite increased antigen cross-presentation. (A) Illustration of the workflow to assess the activation status and frequencies of antigen-presenting cells (APCs) and OVA-specific CD8^+^ T cells in tumors, draining lymph nodes (dLNs), and spleen of B16.OVA-Ctrl or B16.OVA-βA tumor-bearing mice (top), and the influence of anti-CD8 vs anti-CD4 depletion on tumor growth (bottom). (B) H2Kb surface expression and (C), H2Kb-SIINFEKL presentation by CD11c^+^ APCs in B16.OVA-Ctrl and B16.OVA-βA tumors analyzed by flow cytometry. (D) Dextramer-labeled H2Kb-SIINFEKL complexes on CD8^+^ T-cells from B16.OVA-Ctrl and B16.OVA-βA tumors. Error bars, SEM (n=4); *p<0.05, Student’s t-test. (E) MHCII and (F) CD80 expression on tumor-infiltrating CD11c^+^ cells measured as mean fluorescence intensity (MFI) by flow cytometry (n = 8–10). Data represent SEM, ****p<0.0001, Student’s t-test. (G) Growth curves of B16.OVA-Ctrl versus -βΑ in mice treated with IgG, αCD4, or αCD8 antibodies, or with a combination of αCD4 and αCD8. Error bars, SEM (n = 4–5 per group), *p<0.05, **p<0.01, Student’s t-test. ns, not significant.

CTL activation and recruitment can be forestalled if cancer cells downregulate tumor antigen presentation by MHC class I molecules. To address whether Activin-A facilitates this process, we introduced control or *INHBA* lentivirus in B16.OVA mouse melanoma cell cells, a widely used B16-F0 subclone that has been engineered to present the ovalbumin (OVA) epitope SIINFEKL as a surrogate antigen. Flow cytometry of B16.OVA-βA and -Ctrl cells treated with or without IFNγ revealed that cell surface H2Kb (MHC-I) and H2Kb-SIINFEKL complexes were not changed by transgenic *INHBA* (online supplemental figure S2A). Analogous results were obtained in B16F1-Ctrl and -βA cells that we transduced to express increased levels of OVA (online supplemental figure S2B). These results indicate that Activin-A does not interfere with IFNγ-inducible MHC-I loading. To address whether Activin-A inhibits MHC-I antigen cross-presentation, we analyzed intratumoral CD11c^+^ antigen-presenting cells (APCs) and their ability to cross-present OVA to CTLs in B16.OVA tumors (figure 2A). The frequency of intratumoral APCs was comparable in B16.OVA-βA and -Ctrl (figure 2B), and Activin-A increased H2Kb-SIINFEKL cell surface expression, rather than diminishing it (figure 2C). By contrast, the percentage of APCs, CD11c^+^ CD8^+^ cells, and H2Kb-SIINFEKL^+^ subsets and their MHC-I expression in draining lymph nodes (dLNs) remained unchanged (online supplemental figure S2C–F). To test whether the enrichment of intratumoral APCs affects CTL priming, we stained the OVA-specific TCR of CD8^+^ T cells using SIINFEKL-specific MHC-I dextramer. Interestingly, OVA-specific CTLs were enriched 3-fold in βA compared with Ctrl tumors (figure 2D). Furthermore, while MHC-II expression on APCs was unchanged, their expression of co-stimulatory CD80 increased (figure 2E and F). These results suggest that Activin-A secretion by B16 melanoma cells does not promote tumor growth by inhibiting antigen-specific CTL priming.

### Activin-A inhibits CTLs independently of suppressive CD4^+^ T cells

To clarify how Activin-A promotes immune escape, we depleted CD8^+^ or CD4^+^ T cells in βΑ and Ctrl B16.OVA tumor-bearing mice. Selective cell depletion was confirmed in the blood and in tumors (online supplemental figure S2G, H). CD8^+^ T cell ablation with and without CD4^+^ T cell ablation further accelerated the growth of control tumors and neutralized the βΑ-induced growth advantage. By contrast, anti-CD4 treatment induced regression of B16.OVA-Ctrl tumors, but failed to slow the growth of B16.OVA-βA grafts (figure 2G). These data show that Activin-A overall inhibits tumor control by CTLs even independently of CD4^+^ T cells.

### Activin-A does not directly inhibit CTL proliferation or cytotoxicity

**Figure 3:**
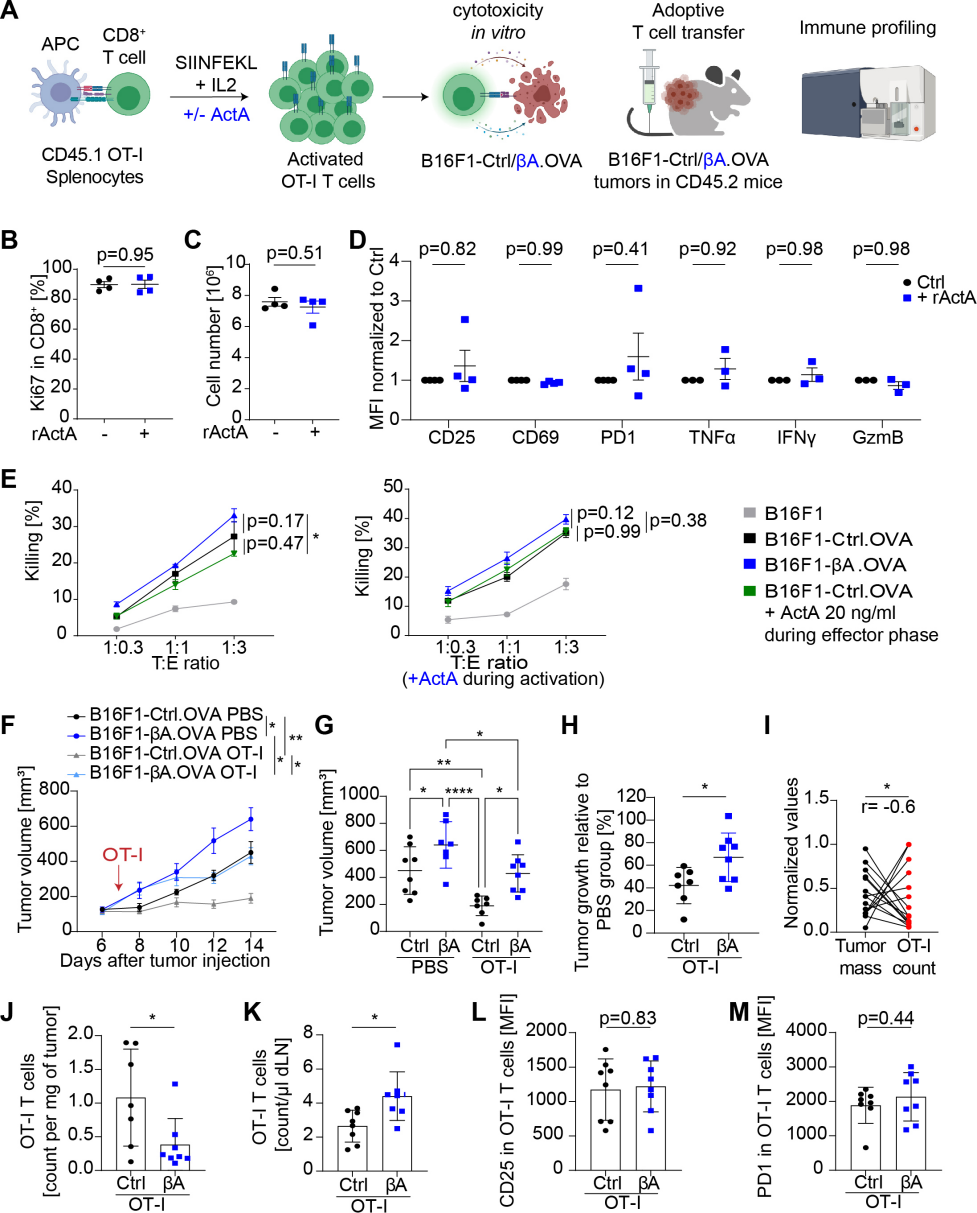
Activin-A diminishes the intratumoral accumulation but not the activation of in vitro-stimulated adoptive T cell grafts. (A) Strategy to compare the in vitro activation and cytotoxicity of CD8^+^ T cells in the presence and absence of melanoma cell-derived or exogenously added Activin-A. (B) Percentage of Ki67^+^ OT-I cells and (C) cell number count of expanded OT-I T cells after activation without or with Activin-A (n = 4 independent experiments). (D) Expression of CD25, CD69, and PD1 (n = 4 experiments) and TNFα, IFNγ, GranzymeB on OT-I T cells (n=3 experiments) after 4 days activation with or without Activin-A analyzed as mean fluorescence intensity normalized to the corresponding control of each flow cytometry experiment. Error bars, SEM; p values, two-way ANOVA with Tukey’s correction for multiple comparison. (E) Tumor cell killing by OT-I cells activated in control conditions (left panel) or in presence of Activin-A (right panel) (n = 3-6 independent experiments). Error bars, SEM, p values, two-way ANOVA with Tukey’s correction for multiple comparison. (F) Growth curves of B16F1-Ctrl.OVA and –βA.OVA tumors in syngeneic mice that received 1x10^6^ activated OT-I T cells or phosphate buffered saline (PBS) by i.v. injection, and (G), tumor volumes measured at the endpoint. (H) Reduction of B16F1-Ctrl.OVA and B16F1-βA.OVA tumor volumes by the OT-I transfer relative to average tumor volumes of the corresponding PBS-treated controls. Error bars, SEM (n = 7–8); p values, ordinary one-way ANOVA with Holm-Šídák correction for multiple comparisons. (I) Correlation of tumor mass with the number of OT-I TILs per mg of tumor. Tumor mass and OT-I count were normalized to the respective values of the largest tumor; n=15; r, two-tailed Pearson correlation analysis. (J) OT-I T cell count per mg of B16F1-Ctrl.OVA and B16F1-βA.OVA tumors and (K)per µl in their dLN suspension. Error bars, SEM (n = 7–8); p values, Student’s t-test. (L, M) Mean fluorescence intensity of CD25 and PD1 protein staining in OT-I T cells analyzed by flow cytometry 1 week after the transfer into B16F1-Ctrl.OVA or B16F1-βΑ.OVA tumor-bearing mice. Error bars, SEM (n=7-8); *p<0.05, **p<0.01, ****p<0.0001, Student’s t-test. ANOVA, analysis of variance; TILs, tumor-infiltrating leucocytes.

To assess whether Activin-A can directly modulate CTL proliferation or function, splenocytes from OT-I mice expressing OVA-specific T cell receptor were stimulated with the OVA peptide SIINFEKL alone or together with Activin-A, and co-cultured thereafter with OVA-expressing melanoma cells (figure 3A). Real-time PCR analysis of total mRNA from treatment-naïve CD8^+^ cells of OT-I transgenic spleen, and from CD8 cell-depleted splenocytes revealed that both populations similarly transcribe *Acvr2a*, *Acvr2b,* and *Alk4*, but no *Alk7* (online supplemental figure S3A). However, treatment with Activin-A during 2 hours, or during OT-I cell activation for 4 days, or thereafter for 2 hours did not increase Smad2 phosphorylation, unlike control treatments with TGFβ (online supplemental figure S3B–D). Moreover, immunostaining of Ki67 and quantification of expanded OT-I cells revealed that SIINFEKL treatment stimulated the proliferation of splenocytes irrespective of Activin-A (figure 3B, C). Activin-A treatment also did not change the expression of the activation markers CD25, CD69, or PD1, or the intracellular staining of TNFα, IFNγ, or Granzyme B (figure 3D, online supplemental figure S3E). These data and analogous treatments with up to 100 ng/mL show that CTLs withstand even high Activin-A dosage.^26^ To assess whether Activin-A inhibits CTLs when it is secreted by melanoma cells, we cocultured activated OT-I T cells with melanoma cells. Since only 10%±5% of B16.OVA cells were killed by OT-I cells in vitro (online supplemental figure S3F), we used B16F1-Ctrl and B16F1-βA cells that we transduced with higher levels of OVA than those detected in B16.OVA cells (online supplemental figure S2B). Neither transgenic *INHBA* nor treatment with recombinant Activin-A before or after activation reduced CTL cytotoxicity (figure 3E). These results strongly suggest that melanoma-derived Activin-A inhibits CTLs indirectly.

### Activin-A impairs tumor infiltration by adoptive T cell transfers

To distinguish whether Activin-A promotes CTL exclusion or exhaustion, we adoptively transferred activated CD45.1 OT-I T cells into CD45.2 tumor-bearing mice. OT-I T cell transfer, but not PBS control, halted the growth of B16F1-Ctrl.OVA tumors. By contrast, B16F1-βΑ.OVA tumors showed only a transient response to adoptive OT-I cell transfer (figure 3F–H). OT-I cell density inversely correlated with tumor size in all tumors analyzed; however, their overall number decreased 2.8-fold in βΑ tumors compared with Ctrl (figure 3I, J), even despite their increase in dLNs (figure 3K). Moreover, OT-I infiltration was accompanied by increased numbers of endogenous CTLs and other leucocytes specifically in B16F1-Ctrl.OVA tumors, suggesting increased inflammation compared with Activin-secreting tumors (online supplemental figure S3G–J). By contrast, OT-I cell activation marked by CD25, PD1, IFNγ, GzmB, and ΤΝFα expression was similar in both groups (figure 3L and M, online supplemental figure S3K–M). These data show that Activin-A impairs intratumoral accumulation of CTLs even after their prior in vitro activation, consistent with an indirect inhibitory mechanism mediated by the TME.

### Immune surveillance and tumor growth are regulated by INHBA in BRAF-driven melanoma

**Figure 4:**
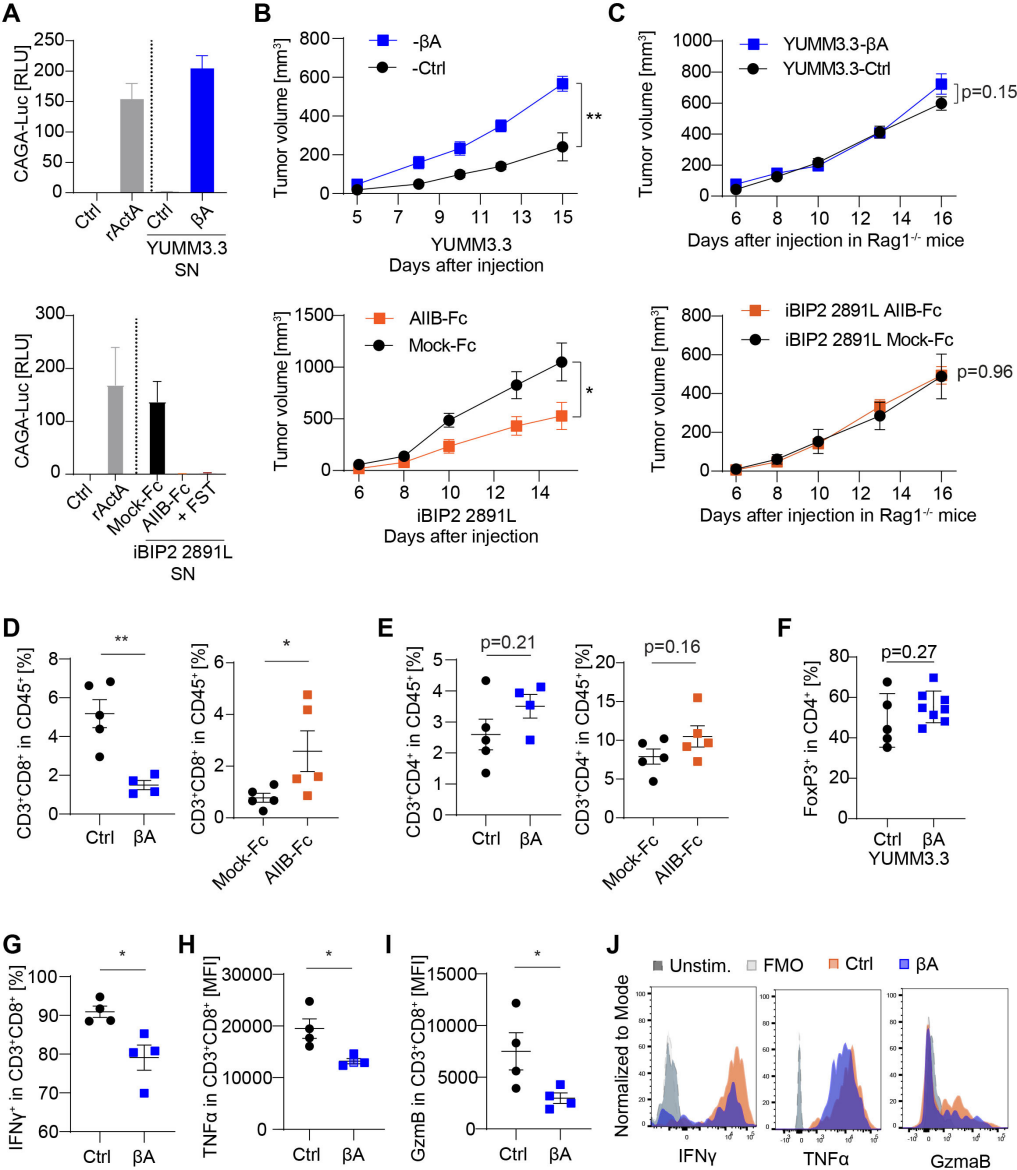
Activin-A accelerates the tumor growth and inhibits CTL accumulation and function also in BRAF-driven melanoma. (A) Induction of the CAGA-luc reporter of SMAD3 activity in HepG2 reporter cells after overnight incubation with conditioned media of YUMM3.3-Ctrl or –βΑ stable cell lines (top), or of iBIP2 2891L Mock-Fc or AIIB-Fc cells (below). SN of parental iBIP2 2891L cells supplemented with or without 100 ng/ml FST are shown for comparison; n=2 independent experiments using triplicate samples. (B) Growth curves of YUMM3.3-Ctrl and –βΑ tumors in syngeneic C57BL/6J female hosts (top), and of iBIP2 2891 L Mock-Fc or AIIB-Fc tumors in syngeneic FVB males (below) (n = 5 per group). (C) Growth curves of YUMM3.3-Ctrl and –βA (top), and iBIP2 2891 L Mock-Fc and AIIB-Fc (below) tumor grafts in Rag1^-/-^ host (n=5 per group). (D, E) Flow cytometry of CD8^+^ or CD4^+^ T cell frequencies in YUMM3.3-Ctrl and –βA (left), iBIP2 Mock-Fc and AIIB-Fc (right) tumor-infiltrating CD45^+^ cells (n=5). Error bars, SEM (n=5 per group); p value, Student’s t-test. (F) Frequency of FoxP3^+^ cells among CD4^+^ TILs in YUMM3.3-Ctrl vs –βA tumors. Error bars, SEM (n = 5 Ctrl and 8 βA tumors), Student’s t-test. (G) IFNγ, (H) TNFα and (I) GzmB expression in CD8^+^ T cells from YUMM3.3-Ctrl and -βΑ tumors at the endpoint. Error bars, SEM (n = 4-5); *p<0.05, **p<0.01, Student’s t-test. (J) Representative histograms of intracellular IFNγ, TNFα, and Granzyme B flow cytometry analysis of CD8^+^ T-cells from YUMM3.3-Ctrl and -βA syngeneic grafts after PMA/ionomycin stimulation. Stained unstimulated cells were used as fluorescence minus one (FMO) staining controls. CTLs, cytotoxic T lymphocytes; TILs, tumor-infiltrating leucocytes.

B16 mouse melanomas do not recapitulate common genetic mutations in human disease and are resistant to immunotherapies.^27^ The most frequent melanoma driver mutations are oncogenic *BRAF*^V600E^ and loss of CDKN2A which are recapitulated in 2891L melanoma cells from *Tyr-CreERT2; Rosa26-lsl-rtTA; tetO-Braf*^V600E^; *Cdkn2*^-/-^; *Pten*^-/-^ iBIP2 mice,^28^ and in YUMM3.3 cells from *Braf*^V600E/+^; *Cdkn2a*^-/-^ mouse melanoma.^29^ RT-qPCR and Western blot analyses revealed that both cell lines transcribe endogenous *Inhba* and accumulate Activin-A protein in the supernatant (SN) (online supplemental figure S4A, B). To monitor Activin-A signaling, we incubated the melanoma cell SNs on HepG2 reporter cells that were stably transduced with the SMAD3 luciferase reporter CAGA-Luc and with Renilla for signal normalization.^30^ Treatment with iBIP2 2891L SN stimulated CAGA-Luc expression more than 100-fold, and this effect was blocked on addition of the Activin-A antagonist Follistatin (FST). By contrast, Activin-A present in YUMM3.3 SN failed to signal and even blocked CAGA-Luc induction by recombinant Activin-A and by iBIP2 2891 L conditioned medium (figure 4A), correlating with high mRNA expression levels of endogenous *Fst* (online supplemental figure S3C–E). To titrate out Fst and possibly other Activin-A antagonists, we transduced YUMM3.3 cells with *INHBA* lentivirus (YUMM3.3-βA) or empty vector (YUMM3.3-Ctrl). Conversely, transduction of iBIP2 2891 L cells with Fc alone (iBIP2-Mock-Fc) or with the ligand trap AIIB-Fc (iBIP2-AIIB-Fc) served to mimic Activin-neutralizing factors and to test the role of endogenous Activin-A. As expected, the conditioned medium of YUMM3.3-βΑ induced CAGA-Luc more than 200-fold (figure 4A, B, C, top panel), whereas AIIB-Fc expression abrogated its induction by iBIP2 cell SN (figure 4A, bottom panel). Moreover, *INHBΑ* expression in YUMM3.3 cells accelerated tumor growth in syngeneic recipient mice, whereas expression of AIIB-Fc in iBIP2 2891L cells inhibited it (figure 4B), even though cell viability in culture was unchanged (online supplemental figure S4F).

To validate whether *INHBA*-induced tumor growth is mediated by the inhibition of adaptive antitumor immunity across multiple melanoma models, we transplanted tumor cells into immunodeficient *Rag1*^-/-^ mice. YUMM3.3 and iBIP2 cells secreting functional Activin-A in *Rag1*^-/-^ hosts grew indistinguishably from their respective Activin-depleted controls, confirming a critical role of adaptive immunity in *INHBA*-induced tumorigenesis (figure 4C). To assess how Activin-A remodels the TME in *Braf*^V600E^-driven melanomas, we profiled TILs using 16-color flow cytometry. While Activin-A overexpression depleted CTLs in YUMM3.3 tumors, expression of ligand trap in iBIP2 2198L grafts increased it, and with no corresponding changes in CD4^+^ T cell frequencies (figure 4D, E). Moreover, in agreement with B16F1 tumor profiling, the frequency of FoxP3^+^CD4^+^ Treg cells was comparable in YUMM3.3-βΑ and -Ctrl tumors (figure 4F). Additionally, intracellular cytokine staining in YUMM3.3 tumor-infiltrating CTLs revealed a marked *INHBA*-induced decrease in IFNγ, TNFα, and GzmB protein levels (figure 4G–J). Collectively, these data demonstrate that Activin-A inhibits antitumor CTLs in both *BRAF* wild-type and mutant melanoma models, and without increasing the frequency of Tregs.

### Activin-A inhibits CTLs independently of CSF1R^+^ TAMs

Analogous to B16F1 tumors, immune profiling revealed that the proportion of CD11b^+^ myeloid cells, but not Ly6C^hi^ monocytes, increased both in YUMM3.3 and iBIP2 βΑ-secreting tumors (online supplemental figure S5A, B). Specifically, *INHBΑ* expression increased the frequency of F4/80^hi^ macrophages in YUMM3.3, or CD11c^+^ F4/80^-^ DCs in iBIP2-Mock-Fc compared with AIIB-Fc, respectively (online supplemental figure S5C, D). To assess the contribution of TAMs to *INHBA*-driven tumor growth, we treated YUMM3.3 tumor-bearing mice with anti-mouse colony stimulating factor 1 receptor (CSF1R) antibodies or isotype controls. Administration of anti-CSF1R antibody failed to significantly alter tumor growth or change the CTL infiltration (online supplemental figure S5E, F), despite a pronounced decrease in the average frequency of tumor-infiltrating F4/80^hi^ TAMs by 61% in YUMM3.3-Ctrl, or by 86% in -βΑ tumors, respectively (online supplemental figure S5G). In good agreement, the expression of IFNγ and TNFα in CD8^+^ and CD4^+^ TILs also remained unchanged (online supplemental figure S5H–K). These data suggest that F4/80^hi^ TAMs do not mediate *INHBA*-induced melanoma growth, or that they function redundantly with cells that are resistant to anti-CSF1R treatment.

**Figure 5:**
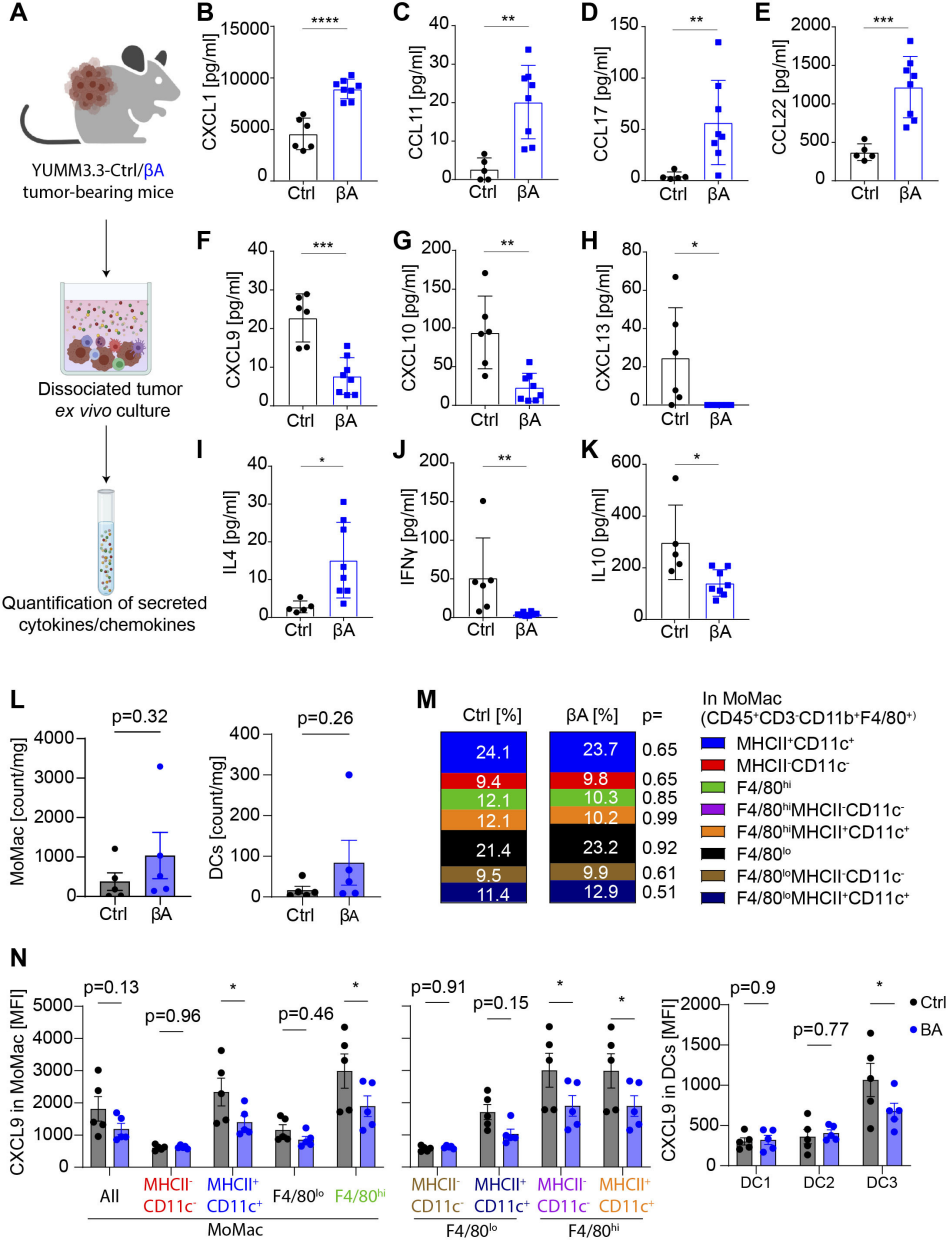
Activin-A alters the immunoregulatory network in the TME. (A) Illustration of the experimental procedure: YUMM3.3-Ctrl and -βΑ tumors were dissected, cell numbers were normalized and cultured ex vivo. Secreted chemokines and cytokines in the culture supernatant were quantified. (B–K) Secretion of CXCL1, CCL11, CCL17, CCL22, CXCL9, CXCL10, CXCL13, IL4, IFNγ, and IL10 by dissociated YUMM3.3-CTRL and YUMM3.3-βΑ tumor cells during 24 hours of ex vivo culture. Error bars, SEM (n = 5-6 Ctrl and 8–βΑ tumors). *P<0.05, **p<0.01, ***p<0.001, ****p<0.0001, Mann-Whitney U test for non-parametric data and a two-tailed t-test for parametric data. (L) Quantification of MoMac (CD45^+^CD3^-^CD11b^+^F4/80^+^) and DC (CD45^+^CD3^-^ F4/80^-^MCHII^+^CD11c^+^) infiltration and (M) proportions of MoMac subsets in YUMM3.3-Ctrl versus -βΑ tumors (n = 5). (N) CXCL9 production measured as MFI in MoMac and DC subpopulations: XCR1^+^ DC1, CD172a^+^ DC2, CCR7^+^ DC3. Error bars, SEM (n=5 tumors per group). *P<0.05, two-tailed t-test. DC, dendritic cell; MFI, mean fluorescence intensity; MHC, major histocompatibility complex; TME, tumor microenvironment.

### Activin-A alters the immunoregulatory network in the TME

Since Activin-A failed to directly inhibit CTL functions in vitro, we sought to profile effects on relevant cytokines and chemokines in cultures of dissociated YUMM3.3-Ctrl and -βΑ tumors (figure 5A). We found that compared with control, βA tumors increased the secretion of CXCL1, CCL11, CCL17, and CCL22, whereas CXCL9, CXCL10, and CXCL13 were decreased (figure 5B–H). Activin-A treatment also reduced CXCL9 and CXCL10 protein secretion by LPS-activated immortalized cDC1 cells in the absence but not in presence of the ALK4 inhibitor SB431542, and without altering cDC1 cell proliferation (online supplemental figure S6A-F). By contrast, the protein levels of secreted CCL2, CCL5, CXCL5, CCL3, CCL4, and CCL20 were comparable in YUMM3.3-βΑ and YUMM3.3-Ctrl tumors (online supplemental figure S7A–F). Dissociated βΑ tumors also secreted more IL4, which promotes Th2 responses and the polarization of suppressive macrophages, whereas IFNγ and IL10 secretion decreased compared with controls (figure 5I–K). By contrast, a tendency for increased IL5 and IL2 secretion did not reach significance, and levels of IL6, TNFα, IL9, IL21, IL13, IL22, IL17A, and IL17F were comparable between both groups (online supplemental figure S7G–P). Therefore, and since we observed no significant increase of *Tgfb1* mRNA expression (online supplemental figure S7Q) or of FoxP3^+^CD4^+^ Tregs in βΑ tumors, we wondered whether Activin-A stimulates tumor growth by promoting IL4 signaling. However, treatment with anti-IL4 antibody did not significantly slow the growth of YUMM3.3-Ctrl or -βA tumors compared with isotype controls (online supplemental figure S7R). Thus, while melanoma cell-derived Activin-A supports myeloid cell infiltration at the expense of CTL recruitment to the tumor site, IL4 is dispensable for Activin-A-induced tumor growth advantage.

**Figure 6:**
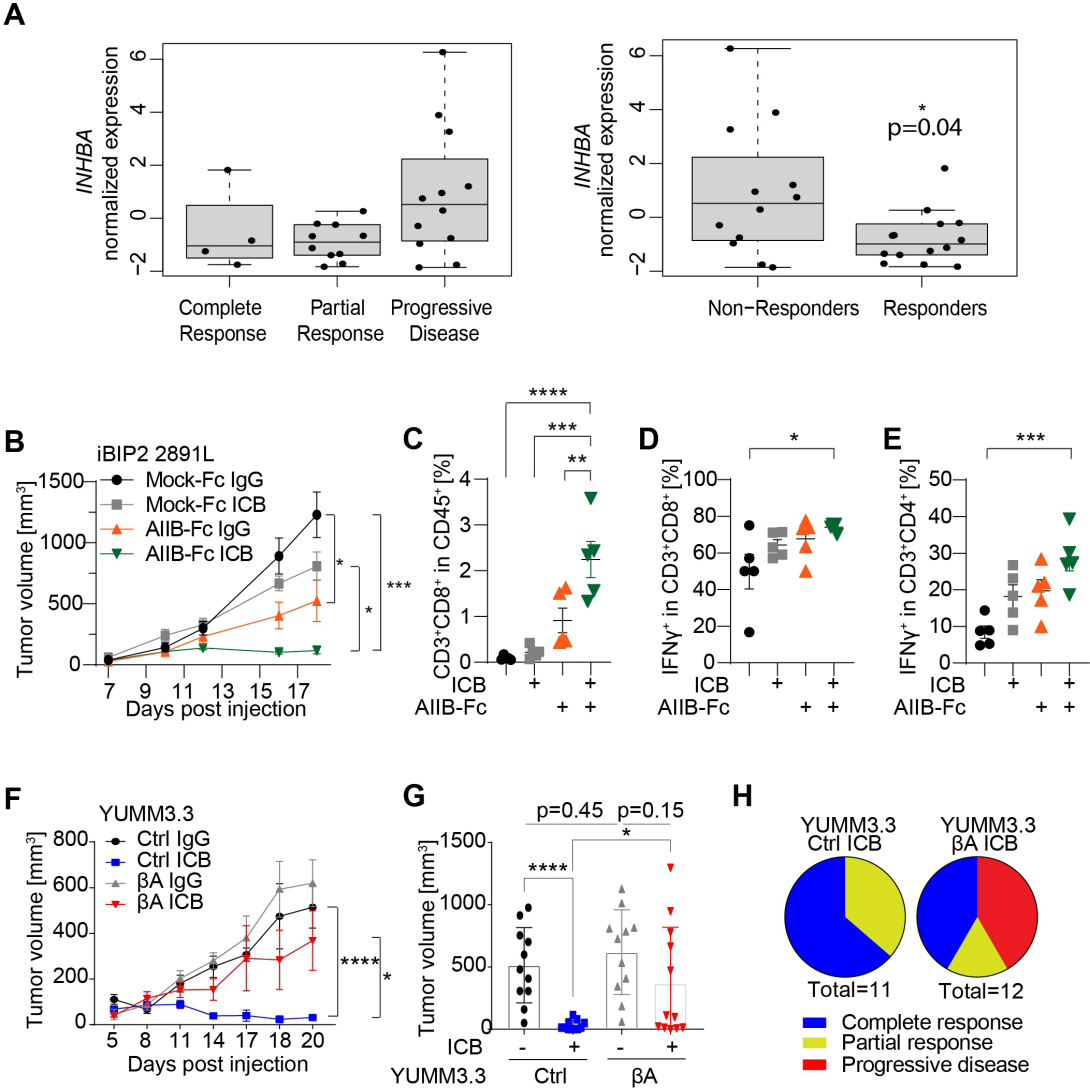
*INHBA* expression in melanoma promotes resistance to ICB immunotherapy. (A) Relative expression levels of *INHBA* mRNA in biopsies of 26 pretreatment melanoma patients grouped by the course of disease after receiving anti-PD1 therapy (left; one-way ANOVA not significant), or as responders (n=14) or non-responders (n=12), p=0.04, Welch two sample t-test (2) based on irRECIST criteria (3). (B) Growth curves of iBIP2 2891L Mock-Fc and iBIP2 2891L AIIB-Fc tumor grafts in FVB/N mice after treatment with αPD-1 and αCTLA4 (ICB), or with IgG control antibodies. (C–E) Relative frequencies of (C) total and (D) IFNγ^+^ CD8^+^ T cells, and of (E) IFNγ^+^ CD4^+^ T cells in iBIP2 2891L Mock-Fc vs AIIB-Fc tumors treated with ICB or control IgG antibodies. Error bars, SEM (n=5 per genotype); **p<0.01, ***p<0.001, ****p<0.0001, ordinary one-way ANOVA with Holm-Šídák correction for multiple comparisons. (F) Growth curves and (G), volumes of YUMM3.3-CTRL and -βΑ tumors treated with IgG or ICB antibodies. (H) Pie charts of the response to anti-PD1/anti-CTLA4 ICB therapy in YUMM3.3-CTRL and YUMM3.3-βΑ tumors. Error bars, SEM (n=11-12); *P<0.05, **p<0.01, ***p<0.001, ****p<0.0001, two-tailed Student’s t-test. ANOVA, analysis of variance.

Secretion of CXCL9 and CXCL10 by myeloid cells is critical for CTL recruitment and tumor control after adoptive T cell transfer or ICB treatment.^31–33^ Absolute quantification of F4/80^+^ monocytic macrophage (MoMac) or DCs showed no change in YUMM3.3-βΑ compared with YUMM3.3-Ctrl tumors, and proportions of MoMac subtypes were comparable between the two groups (figure 5L and M). However, in both MHCII^+^CD11c^+^ and F4/80^hi^ MoMac, and CCR7^+^ DC3 cells, *INHBA*-expressing tumors showed significantly reduced intracellular staining of CXCL9 (figure 5N, online supplemental figure S7S). Together, these data indicate that Activin-A expression in tumor interferes with CTL recruitment by attenuating the expression of CXCL9 in MoMac and DCs.

### *INHBA* expression in melanoma augments their resistance to ICB immunotherapy

Analysis of public datasets revealed that *INHBA* mRNA expression in human melanoma only weakly correlates with survival^15^ or metastatic burden (online supplemental figure S8A). Average *INHBA* mRNA levels in the TCGA dataset of human melanoma (SKCM) were also comparable in primary and metastatic tumors (online supplemental figure S8B). Interestingly, however, in a published dataset on genomic and transcriptomic changes from 28 melanoma patients treated with anti-PD1, immunotherapy resistance strongly correlated with *INHBA* expression (figure 6A).^34^ Innate resistance to immunotherapies by anti-PD1, anti-CTLA4, or anti-CSF1R antibodies is also seen in iBIP2 mouse melanoma.^28^ To test whether resistance involves endogenous Activin-A, we administered anti-PD1/anti-CTLA4 immune checkpoint blockade (ICB) therapy to iBIP2-AIIB-Fc or iBIP2-Mock-Fc tumor-bearing mice. While ICB administration failed to significantly inhibit iBIP2-Mock-Fc tumor growth, it led to uniform regression of AIIB-Fc tumors (figure 6B). FACS analysis of TILs revealed that this response to ICB was accompanied by increased CTL infiltration and by a higher frequency of CD8^+^ and CD4^+^ T cells expressing IFNγ (figure 6C–E). To test whether *INHBA* is both necessary and sufficient to confer ICB resistance, we administered anti-PD1/anti-CTLA4 ICB therapy or IgG control antibodies to YUMM3.3-Ctrl and -βΑ tumor grafts. As expected, YUMM3.3-Ctrl tumors invariably responded to ICB therapy, with 64% of the tumors achieving complete regression (n=7/11) and 36% partial response (n=4/11). By contrast, a majority of YUMM3.3-βΑ tumor-bearing mice showed progressive disease (n=5/12, 42%) or only a partial response (n=2/12, 17%) despite ICB treatment (figure 6F–H, online supplemental figure S8C). Together, these results demonstrate that *INHBA*-induced changes in the TME impair the response of melanoma to ICB therapy.

## Discussion

Here, we found that Activin-A indirectly inhibits CTL proliferation and accumulation in *BRAF* wild-type and oncogenic mutant preclinical melanoma models and that its expression correlates with anti-PD1 therapy resistance in human melanoma and impairs the response to combined anti-PD1/anti-CTLA4 therapy in gain-of-function and loss-of-function settings. Immune-profiling and cell depletion experiments revealed that melanoma cell-derived Activin-A reduces the dependence on suppressive CD4^+^ T cells to escape immune surveillance, correlating with the diminished secretion of specific chemokines such as CXCL9 and CXCL10. Our findings suggest that Activin-A neutralization is a promising strategy to sensitize *INHBΑ*-expressing tumors to ICB therapy.

In all melanoma models examined, and irrespective of their *BRAF* status, *INHBA* expression reduced the frequency of intratumoral CTLs and NK cells. Activin-A can attenuate NK cell proliferation and cytotoxicity directly, although modestly.^26 35^ In addition, NK cells are likely depleted due to impaired accumulation of CTLs.^36 37^ Indeed, several lines of evidence suggest that immune evasion induced by Activin-A requires reprogramming of the TME, which in turn primarily inhibits the recruitment and activity of CTLs: First, the tumor growth advantage mediated by Activin-A was lost in *Rag1*^-/-^ hosts that specifically lack adaptive immunity. Second, Activin-A secretion by melanoma cells stimulated T cell priming rather than inhibiting it, and only locally within the TME, even though it accumulates in the circulation and induces systemic muscle wasting. Third, Activin-A diminished tumor infiltration of in vitro-activated OVA-specific adoptive T cell grafts, and despite the fact that they maintained the expression of activation markers and of cytotoxic effector molecules for at least 1 week after adoptive transfer into tumor-bearing mice. By contrast, Activin-A treatment of OVA-specific T cells in vitro altered neither their proliferation nor their cytotoxicity. Accordingly, Activin-A also failed to stimulate Smad2 phosphorylation in activated CD8^+^ T cells and in naïve splenocytes expressing *Acvr2* and *Alk4* mRNAs. Possible inhibitory mechanisms include the expression of specific antagonists or post-transcriptional regulation of Activin receptors. Our finding that Activin-A only indirectly inhibited CD8^+^ T cell proliferation agrees with earlier reports that it does not alter the proliferation of CD8^+^ T cells or their IFNγ expression, or that it may do so only indirectly.^23 38^ Lastly, analysis in B16.OVA melanoma revealed that depletion of CD8^+^ T cells abolishes the tumor-promoting activity of *INHBA*. By contrast, anti-CD4 depletion only diminished the growth of B16.OVA-Ctrl but not of -βA tumors. Thus, Activin-A reduces the dependence of these tumors on suppressive CD4^+^ T cells to escape immune detection, rather than increasing it. Lack of Tregs in *Cd4*^-/-^ hosts also does not slow *INHBA*-driven growth of a papilloma virus-induced skin cancer model.^18^ At first glance, this is surprising since Activin-A can synergize with TGFβ to induce Tregs.^39 40^ However, Tregs only express Alk7, which preferably binds *INHBB*-derived Activins.^24^ The failure of melanoma cell-derived Activin-A to enrich Tregs or to increase CD4^+^ T cell-mediated immune evasion in any of our preclinical models points to its limited bioavailability in this cell type.

Cancer cells can evade CTLs by reducing tumor antigen presentation, and inefficient MHC-I induction by IFNγ in melanoma correlates with resistance to ICB therapy.^4^ Our analysis revealed that *INHBA* expression attenuated the levels of IFNγ protein in bulk tumors and in infiltrating CTLs, yet without altering MHC-I expression by tumor cells. Another pillar of antitumor T cell response involves the cross-presentation of tumor antigens by DCs. Treatment with Activin-A can inhibit or promote CD8^+^ T cell activation by in vitro-differentiated mouse and human DCs, depending on the context.^23 41^ Our analysis revealed that the majority of intratumoral APCs are CD11b^+^ myeloid cells. Activin-A increased their antigen presentation rather than inhibiting it, and without altering their overall frequency or their migration to dLNs, or their MHC-I or -II expression. Such efficient cross-presentation even despite the presence of Activin-A sharply contrasts the potent inhibition of DCs by Activin-induced Tregs in allergic airway disease,^42^ consistent with our finding that melanoma cell-derived Activin-A did not enrich Tregs. Furthermore, instead of activating CTLs, the stimulation of cross-presenting APCs by Activin-A associated with reduced CTL proliferation and effector functions. Thus, besides inhibiting CTL recruitment, Activin-A reprograms the TME to interfere with their proper activation.

In all melanoma models examined, the depletion of lymphocytes by Activin-A was accompanied by a proportionate increase in myeloid infiltrate, but its composition varied considerably. In B16-F1 melanoma, Activin-A promoted the recruitment of Ly6C^hi^ monocytes without further enriching TAMs. By contrast, in iBIP2 and YUMM3.3 grafts, it mainly enriched TAMs, but not monocytes. Whether such differences in the composition of myeloid infiltrates and their relative enrichment by Activin-A depend on BRAF status or other tumor-specific driver mutations is unknown. However, macrophages are also enriched by a keratinocyte-specific *INHBA* transgene in HPV8-induced skin tumors, and their depletion by anti-CSF1R treatment delayed the onset of tumor formation.^18^ By contrast, we found that depletion of CSF1R^+^ TAMs did not reduce *INHBA*-induced melanoma growth. In good agreement, CSF1R antibody depletion also failed to slow tumor growth in iBIP2 transgenic mice or in mice receiving Yummer1.7 melanoma cell transplants.^28^ Myeloid-derived suppressive cells can also be recruited by the chemokine receptor CCR2 that is induced by Activin-A in monocyte-derived macrophages.^43^ Interestingly, CCR2 inhibition can boost ICB therapies in preclinical models of several cancer types.^44^ However, analysis in B16-F10 melanoma-bearing mice revealed that CCR2 inhibition depleted only a prometastatic subpopulation of lung-resident macrophages without diminishing primary tumor growth.^44^ Similarly, genetic ablation of CCR2^+^ monocytes also afforded no protection against *INHBA*-driven skin tumors or against their infiltration by tumor-promoting TAMs.^18^

Tumor control after adoptive T cell transfer and the response of melanoma to immune checkpoint therapy depend on the IFNγ-inducible chemokines CXCL9/10 to recruit CXCR3-expressing T cells to the tumor bed.^32 45 46^ CXCL9 and CXCL10 expression also correlate with T cell infiltration and survival in stage-III melanoma patients.^47–49^ However, little is known about negative regulators of CXCL9/10 expression in cancer. Our chemokine profiling of dissociated YUMM3.3 tumors revealed that Activin-A expression diminished IFNγ and CXCL9/10 secretion. In vivo validation of CXCL9 expression confirmed its downregulation both in CCR7 +DCs and in MHCII^+^CD11c^+^ MoMac and F4/80hi TAMs. Activin-A treatment of a widely used mouse cDC1 cell line during activation by LPS and IFNγ within 24 hours led to a significant decrease in CXCL9 and CXCL10 secretion. Conversely, treatment of monocyte-derived DCs with FST has previously been shown to rapidly increase CXCL10 secretion, indicating direct inhibition also by autocrine Activin signaling.^23^ TAMs expressing CSF1R are not essential to mediate T cell exclusion by Activin-A, since their depletion did not slow *INHBA*-dependent tumor growth. However, alternative CXCL9/10 sources including CSF1R-independent MoMac populations and DCs are critical for CTL recruitment.^32^ In addition, Activin-A increased the levels of secreted CCL11, CCL17, CCL22, and IL4. CCL11 expression is induced by hypoxia in breast cancer cells to recruit CCR5^+^ macrophages,^50^ whereas CCL17 and CCL22 secreted by tumor cells, TAMs, and/or DCs can attract CCR4-expressing CD4^+^ Th17, Treg, and Th2 cells and monocytes with protumor or antitumor activities.^51^ Activin-A secretion by melanoma cells clearly stimulated tumor growth independently of CD4^+^ cells and IL-4. However, the potential roles of DCs and of monocyte recruitment by CCR4 ligands warrant further investigation.

Our analysis of a publicly available dataset indicated that *INHBA* was upregulated in melanoma patients with progressive disease after anti-PD1 therapy.^34^ Furthermore, neutralization of endogenous Activin-A by a soluble form of ACVRIIB sensitized iBIP2 melanoma grafts to combined anti-PD1/anti-CTLA4 blockade, whereas *INHBA* gain-of-function in YUMM3.3 melanoma grafts inhibited the response. In both settings and across different models, ICB response correlated with CTL infiltration and activation as assessed by intracellular IFNγ staining. These results strongly support a clinically relevant function for Activin-A signaling in the resistance to checkpoint inhibitors in melanoma.

10.1136/jitc-2022-004533.supp2 Supplementary data

## Data Availability

Data sharing not applicable as no datasets generated and/or analysed for this study.
