# Supplementary material for: Activin-A impairs CD8 T cell-mediated immunity and immune checkpoint therapy response in melanoma
Source: J Immunother Cancer. 2022 May 16;10(5):e004533. doi: 10.1136/jitc-2022-004533 (PMC9125758; doi:10.1136/jitc-2022-004533)
Supplement: Supplementary data [file jitc-2022-004533supp001.pdf]

SUPPLEMENTARY MATERIALS

Activin-A impairs CD8 T cell-mediated immunity and immune checkpoint therapy response in melanoma

Katarina Pinjusic[1*], Olivier A. Dubey[1*], Olga Egorova[1], Sina Nassiri[1, 2], Etienne Meylan[1, 3, 4, 5], Julien Faget [1, 6], and Daniel B. Constam[1].

[1] Ecole Polytechnique Fédérale de Lausanne (EPFL) SV ISREC, Station 19, CH-1015 Lausanne, Switzerland

[2] Bioinformatics Core Facility, SIB Swiss Institute of Bioinformatics, CH-1015 Lausanne, Switzerland

[3] Laboratory of Immuno-Oncology, Bordet Cancer Research Laboratories, Institut Jules Bordet, Faculty of Medicine, Université Libre de Bruxelles, 1070 Anderlecht, Belgium

[4] Laboratory of Immunobiology, Faculty of Sciences, Université Libre de Bruxelles, 6041 Gosselies, Belgium

[5] ULB Cancer Research Center (U-CRC) and ULB Center for Research in Immunology (U-CRI)

[6] Present address: IRCM, Inserm, Univ Montpellier, ICM, Montpellier, France, INSERM U1194, Montpellier, France

* These authors contributed equally to this work

Corresponding author: Daniel B Constam, +41 21 693 07 41, Daniel.Constam@epfl.ch

SUPPLEMENTARY MATERIALS AND METHODS

SUPPLEMENTARY REFERENCES

SUPPLEMENTARY TABLES

Supplementary Table 1. Activin-A induced changes in the B16-F1 TME

Supplementary Table 2. qPCR primers

Supplementary Table 3. Antibodies used for immune-profiling

SUPPLEMENTARY FIGURES

Supplementary Figure 1. Effects of *INHBA* on the composition, cell proliferation, and gene expression of immune infiltrates in B16-F1 melanoma.

Supplementary Figure 2. Antigen cross-presentation in blood and lymphoid organs and analysis of CD4/CD8 cell depletion in B16.OVA-βA melanoma-bearing mice.

Supplementary Figure 3. Analysis of *in vitro* activated T cells and their effect on tumor immune infiltrates after adoptive transfer in B16-F1 melanoma-bearing mice.

Supplementary Figure 4. Characterization of YUMM3.3 and iBIP2 2891L cell lines.

Supplementary Figure 5. Activin-A accelerates melanoma growth independently of CSF1R$^+$ TAMs.

Supplementary Figure 6. Activin-A directly impairs CXCL9/10 production by immortalized cDC1 cells.

Pinjusic K, *et al. J Immunother Cancer* 2022; 10:e004533. doi: 10.1136/jitc-2022-004533

Supplementary Figure 7. Expression of immune regulators that were not modulated by melanoma-derived Activin-A.

Supplementary Figure 8. Correlation of *INHBA* expression with the survival of melanoma patients.

Supplementary Figure 9. Gating strategy for TIL profiling by a 16-color flow cytometry antibody panel.

Supplementary Figure 10. Gating strategy used to analyze OVA-specific T cells and SIINFEKL presentation by APCs in figures 2 and S2.

Supplementary Figure 11. Gating strategy used for ATCT analyses in Fig. 3 and S3.

Supplementary Figure 12. Gating strategy used in Fig. 5 and S7 to analyze CXCL9 expression in tumors.

Pinjusic K, *et al. J Immunother Cancer* 2022; 10:e004533. doi: 10.1136/jitc-2022-004533

SUPPLEMENTARY MATERIALS AND METHODS

Cell lines

For all cell lines, culture media were supplemented with 10% fetal bovine serum, 50 µg/mL gentamicin (Gibco, Thermo Fisher Scientific, Waltham, MA, USA), and 1% GlutaMAX (Gibco). B16F1, HepG2, and HEK293T cells were purchased from ATCC) and maintained in DMEM (Sigma-Aldrich, St. Louis, MO, USA). YUMM3.3 cells were provided by Dr. Anna Obenauf (Vienna, AU) and maintained in DMEM/F12 (Gibco). iBIP2 cell lines were provided by Mélanie Tichet (Hanahan lab, EPFL, Lausanne, CH) and maintained in RPMI in presence of 1 µg/mL doxycycline (Sigma-Aldrich). B16-F0 cells expressing lentiviral ovalbumin (B16.OVA) have been described [1]. HepG2 reporter cells were established by stable lentiviral transduction of a CAGA-Luc reporter and Renilla luciferase for normalization [2]. All cell lines were regularly tested negative for Mycoplasma (SouthernBiotech, Birmingham, AL, USA, 13100-01 or Biontex, Munich, DE, M020), and authenticated by the presence or absence of pigmentation, cell shapes, and unique responses of characteristic luciferase reporters or Dox-inducible transgenes.

Expression vectors and cloning

A Spe1 fragment of INHBA cDNA (NM_002192.2, nucleotides 218-1609, plus myc tag) was subcloned into XbaI-digested pLenti-EF1alpha-MCS-SV40-puro vector [3]. pLenti-EF1alpha-ActA-SV40-bsd was generated by subcloning INHBA from pLenti EF1a-MycActA SV40puro into pLenti EF1a-OVA bsd. In brief, the INHBA coding sequence was PCR-amplified using the primers 5’-GGGGAGATCTAAGGCAATCACAACAACTTTTGC-3’ and 5’-CACAGGGTCGACCACTGGTC -3’ that introduced a Bgl II site at the 5’ end. The resulting fragment was digested with BglII/MluI and inserted into BglII/MluI-digested pLenti EF1a-OVA bsd. pLenti-EF1alpha-AIIB-Fc-SV40-bsd and pLenti-EF1alpha-Mock-Fc-SV40-bsd were cloned using the same strategy, but using the primers 5’-GGGGAGATCTGCCTCGAGAATTGCTTCCAC-3’ and 5’-GGGTCGACCACTGGT CGAC-3’.

Lentiviral transduction

YUMM3.3-Ctrl and -βA, iBIP2-Mock-Fc and -AIIB-Fc, B16F1-Ctrl.OVA and -βA.OVA cell lines were generated as previously described for B16F1-Ctrl and -βA [3]. In short, HEK293T cells were co-transfected with CMVΔR8.74 (Addgene, Watertown, MA, USA 22036), pMD2.VSVg (Addgene 12259) mycINHβA, AIIB-Fc, OVA or empty transfer plasmid. Lentiviral particles were collected from filtered culture supernatant by ultracentrifugation and resuspended in sterile PBS. Target cells were transduced in a 12-well plate. YUMM3.3, iBIP2, and B16F1 cells were selected using blasticidin.

Reporter assay

HepG2 CAGA-Luc reporter cells [2] were seeded in 96-well plate and treated with 20 ng/mL recombinant Activin-A (R&D Systems, Minneapolis, MN, USA) as a positive control or with supernatant from INHBA- or Ctrl-transduced melanoma cells. Where indicated, 250 ng/mL of Follistatin (R&D Systems) was added to the cell condition medium. After overnight incubation, cell proteins were extracted in potassium phosphate buffer containing 0.5% Triton X-100. Luminescence of Firefly and Renilla luciferase was measured in 5 µL of the extract by Centro LB960 luminometer in a white 96-well plate (Nunc 96 MicroWell™ Plates #236108) by the addition of 50 µL P/R A (Firefly) and P/R B (Renilla) reagents [4]. Relative light units (RLU) represent firefly expression normalized to renilla and expressed relative to the non-treated control.

Cell viability assay

Cells were plated in 96-well plates at a density of $5x10^3$cells/well and cultured for 3 days. Alamar blue reagent (Invitrogen, DAL1025) was added to 90% confluent cells and fluorescence was measured 4 hrs later on a TECAN spectrophotometer at the emission wavelength of 590 nm after excitation at 560 nm.

Western blot analysis

Cells were lysed in a homemade RIPA buffer supplemented with the protease inhibitor cocktail (Roche, Basel, CH) and phosphatase inhibitors (Sigma-Aldrich). The conditioned medium was precipitated using ice-cold acetone and resuspended in RIPA buffer. Protein concentration was

measured by BCA assay (Thermo Fisher Scientific). Proteins were separated on 9-12% SDS-PAGE gels under reducing or non-reducing conditions and transferred on nitrocellulose membranes. Membranes were blocked with 5% skim milk (Sigma) in Tris-buffered saline containing 0.1% Tween-20, before incubation with primary antibodies against phosphorylated or total SMAD2 (Cell Signaling 8828, and 3103), γ-tubulin (Sigma GTU88), GAPDH (Abcam ab70699, Cambridge, UK), Activin-A (Abcam ab89307), Ovalbumin (Milipore AB1225, Burlington, MA, USA) for 2 hrs at room temperature or overnight at 4°C. For fluorescence detection, proteins were visualized on an Odyssey CLx instrument after incubation with a secondary antibody coupled with IRDye 800CW and IRDye 680RD (all from Licor, Lincoln, NE, USA). Chemiluminescence was revealed on X-ray film (Kodak, Rochester, NY, USA) or ChemiDoc MP (Biorad, Hercules, CA, USA) using HRP-coupled secondary antibodies and ECL reagents (Thermo Fisher).

Quantitative polymerase chain reaction

Cells were cultured in 6-well plates and RNA was extracted using the ReliaPrep RNA Cell Miniprep System (Promega, Madison, WI, USA) following the manufacturer's protocol. For total tumor RNA extraction, snap-frozen tumor pieces were sonicated or homogenized in 1 mL of QIAzol (Qiagen, Hilden, DE), and followed by chloroform extraction. Total RNA was isolated using the RNeasy mini kit (Qiagen) according to the manufacturer's protocol. cDNA was synthesized from 1 µg RNA using the PrimeScript RT-PCR Kit (Takara, Kusatsu, JP). The analysis was performed using the SYBR Green GoTaq Master Mix (Promega) on a QuantStudio 6 instrument (Applied Biosystems, Waltham, MA, USA). qPCR primers are listed in **Table S2**.

Melanoma grafts and adoptive T cell transfers

B16F1, B16.OVA, B16F1.OVA, YUMM3.3: 10^5 cells were injected intradermally (B16F1 & B16.OVA) or $2.5x10^5$ cells subcutaneously (YUMM3.3), or $8x10^5$ cells subcutaneously (B16F1-OVA) into the right flank of 8-12 week old female C57BL/6 mice (Charles River laboratories) [1]. Alternatively, 8-12 week old male FVB/N mice (Charles River, Wilmington, MA, USA) received 10^5 iBIP2 2891L cells subcutaneously the day after they were switched to 0.625 g/Kg Doxycycline Hyclat food pellets (Safe, E8404 Version 0002_150) to activate the rtTA-induced

expression of BRAF V600E [5]. Rag1-/- mice were bred at EPFL. For OT-I adoptive T cell transfer, OT-I CD45.1 splenocytes were activated 5 days in vitro as described below. Before the transfer, T cells were extensively washed in PBS and dead cells were removed using the dead cell removal kit (Miltenyi Biotec, 130-090-101, Bergisch Gladbach, DE) following the manufacturers instruction. $1x10^6$ activated OT-I T cells in 100 µl PBS, or PBS alone were injected i.v. per mice on day 7 after the tumor challenge. Tumors were measured 3 times/week and volumes were calculated using the formula $V = [1.58\pi \times (\text{length} \times \text{width})^{3/2}]/6$ [6].

Quantification of Activin-A plasma levels

At the experimental endpoint, blood was collected in heparin-coated tubes (Microvette 500 LH, SARSTEDT AG & Co.). Plasma was separated by centrifugation and aliquoted into new Eppendorf tubes. Activin-A levels in plasma were quantified by ELISA following the manufacturer's instructions (R&D DAC00B).

Antibody injections

To deplete T cells, mice were intraperitoneally injected with 10 mg/kg anti-CD4 (clone YTS191) and/or anti-CD8 (clone YTS169.4), or rat IgG2b (clone LTF-2, all from BioXcell, Lebanon, NH, United States) in 200 µL PBS twice weekly, starting one day before tumor grafting. For anti-CSF1R depletion, treatment of YUMM3.3 was initiated one day before the tumor graft and administered twice weekly thereafter. Mice were treated with 50 mg/kg i.p. rat anti-CSF1R (clone AFS98, BioXCell) or control rat IgG2a antibody (clone 2A3, BioXCell). For anti-PD1 and anti-CTLA4 pre-clinical studies, mice were intraperitoneally injected every 3 days with 10 mg/kg anti-PD1 (clone RMP1-14), 5 mg/kg anti-CTLA4 (clone 9H10), or 10 mg/kg rat IgG2a and 5 mg/kg Syrian hamster IgG (all from BioXcell) in 200 µL PBS. For anti-IL4 injections, mice were treated with 1mg of anti-IL4 (clone 11B11, BioXcell) or rat IgG (clone HRPN, BioXcell) one day before the tumor injections, followed by 500 µg every 5 days. All procedures were according to Swiss legislation and approved by the cantonal veterinary administration.

Tumor dissociation and flow cytometry analyses

Tumors were dissected, minced using rounded scissors, and digested in Dnase I (0.02 mg/mL, Sigma) and collagenase (1 mg/mL, Sigma) in RPMI using a gentleMACS Octo Dissociator

Pinjusic K, *et al. J Immunother Cancer* 2022; 10:e004533. doi: 10.1136/jitc-2022-004533

(Miltenyi). Red blood cells were lysed using PharmLyse buffer (BD Biosciences). Cells were washed with PBS and 1-5x10^6 of cells were used for staining. Cells were incubated with mouse FcR blocking solution (1:200, Miltenyi) and Live/Dead fixable blue dead cell stain (1:1000, Life Technologies, Carlsbad, CA, USA) for 30 min. After washing, cells were stained for surface markers for 45 min in FACS buffer (2% FBS, 2 mM EDTA in PBS). For dextramer staining, 2 µL MHC-I SIINFEKL dextramers (Immudex, Copenhagen, DK) were added before antibody staining. MHC-I SIYRYYGL dextramer was used as a negative control. For intracellular cytokine staining, 10^6 cells were plated in the complete RPMI medium and incubated for 5 hrs with eBioscienceTM Cell Stimulation Cocktail (00-4970-93) and protein transport inhibitor (BD 555029). Cells were then washed and stained for surface markers, followed by fixation and permeabilization using the FoxP3 staining buffer set (eBioscience) before intracellular staining. After the staining, cells were collected in FACS buffer, and data were acquired using an LSRII SORP or an LSR Fortessa cytometer (Becton Dickinson, Franklin Lakes, NJ, USA). Antibodies used for 16-color panel and other stainings are listed below in **Table S3** [7].

To quantify OT-I cells, tumors were weighed, digested, and resuspended in 100 µl PBS per 50 mg of a tumor. Single-cell suspensions of draining lymph nodes were obtained by pressing the lymph node against the mesh of a cell strainer. Dissociated cells were washed and resuspended in 500 µl PBS. 100 µl of tumor and 200 µl of lymph node cell suspension were used for antibody staining. Stained samples were resuspended in 200 µl FACS buffer and loaded on Precision Count Beads (25 µl, BioLegend 424902, San Diego, CA, USA). Data were acquired on an LSR Fortessa cytometer using beads as a stopping gate. OT-I cell numbers were calculated following the manufacturer's instructions. For intracellular CXCL9 staining, tumors were dissociated and single-cell suspensions were incubated for 3h with protein transport inhibitor (BD 555029) without additional stimulation and stained for intracellular CXCL9 as described above for intracellular cytokines. Gating strategies are provided in **Supplementary Figures 9-12.**

In vitro T cell activation and cytotoxicity assay

Splenocytes were collected from OT-I CD45.1 transgenic mice. Red blood cells were lysed using RBC lysis buffer, and 5x10^6 cells/mL activated with 10 µg/mL of SIINFEKL peptide (Sigma

Aldrich S7951) and 10 ng/mL rhIL-2 (Peprotech 200-02, East Windsor, NJ, United States) for 3-5 days in the T-cell medium. Where indicated, cells were activated in presence of 20-100 ng/mL recombinant Activin-A (R&D Systems) or treated with 20 ng/ml TGFβ1 (Gibco). The T-cell medium was prepared by supplementing the RPMI with 10% heat-inactivated FBS, 1% PS, 1 mM sodium pyruvate, 50 µM Beta-ME, 100 µM non-essential amino acids, 10 mM HEPES followed by sterile filtration. To analyze activation markers, OT-I T cells from individual spleens were expanded independently from each other for each experiment and stained with different panels of antibodies, resulting in some variability in baseline fluorescence intensities. Therefore, the signals after Activin-A treatment were normalized to each individual corresponding control. Source data without normalization are shown as Supplementary information. For killing assays, activated T cells were washed and rested for 6 hrs in a medium without cytokines. $1x10^6$ tumor cells/mL PBS were stained with 1 µL/mL Cell Trace CSFE (ThermoFisher C34554) for 20 min at 37°C and washed before overnight co-culture with activated OT-I T cells at the indicated ratios. To quantify the killing of tumor cells by OT-I T cells, co-culture supernatant and adherent cells were collected. Samples were stained using Annexin V-APC Apoptosis Detection Kit with PI (Biolegend 640932) following the manufacturer's protocol and fluorescence signals were acquired using LSRII SORP or an LSR Fortessa cytometer (Becton Dickinson). Tumor cell killing percentage is calculated as = (% of live tumor cells - % of live cells in tumor only control) / % of live cells in tumor only control x -100.

Cytokine and chemokine quantification

Tumors were dissected, minced using rounded scissors, and digested in DNase-I (0.02 mg/mL, Sigma) and collagenase (1 mg/mL, Sigma) in RPMI using a gentleMACS Octo Dissociator (Miltenyi). After washing and counting with a TC20 automated cell counter (Bio-Rad) $5x10^6$ cells/mL were cultured in 96-well plates for 28 hrs in the complete DMEM-F12 medium. Factors secreted into the culture supernatant were cleared from cells and debris by centrifugation and stored at -80°C until analyses. The supernatants were analyzed undiluted or diluted 70x and 13 secreted cytokines and chemokines were analyzed using LEGENDplex™ Mouse Th Cytokine Panel (13-plex) and LEGENDplex™ Mouse Proinflammatory Chemokine Panel (13-plex) (both from BioLegend) following manufacturer's protocol, respectively. Data were

acquired using an LSR Fortessa cytometer (Becton Dickinson) and analyzed using BioLegend's LEGENDplexTM Data Analysis Software (BioLegend).

Mouse cDC1 cell line culture and activation

Immortalized mouse cDC1 cells [8] were cultured in IMDM medium supplemented with 10% heat-inactivated FBS, 100x Glutamax, 10 mM HEPES, 100 μM Pen/Strep, 50 μM β-mercaptoethanol at 37° and 5% CO_2. PBS supplemented with 20 mM HEPES and 5 mM EDTA was used for cell passaging. For activation, 250'000 cells were seeded per well in a 12 well plate and incubated for 24 hrs with 5 μg/ml LPS and 10 ng/ml IFNγ. Where indicated, cells were also incubated with 50 ng/ml recombinant Activin-A, TGFβ, or BMP4 (all from R&D), or 10 μM SB431542 (Tocris Bioscience). During the last 3 hrs of incubation, Golgi Plug (BD 555029) was added to the cells followed by intracellular cytokine staining using the protocol described above. Supernatants were collected for CXCL9 and CXCL10 quantification by ELISA following the manufacturer's instructions (Abcam ab203364 and ab260067, respectively).

Bioinformatics analysis

The association between the expression levels of INHBA mRNA and response to anti-PD1 therapy was assessed using a previously published RNA-seq dataset [9]. Processed data and metadata were obtained from Gene Expression Omnibus (GEO) and visualized in R. One-way ANOVA was not significant when comparing complete response, partial response, and progressive disease samples. By pooling the complete and partial responses into one group, we found a significant difference between responders and non-responders using Welch two sample t-test. Kaplan-Meier survival curves comparing TCGA melanoma patients stratified by the expression level of the INHBA gene were obtained from TIMER database (http://timer.cistrome.org/). To assess the correlation between INHBA expression levels and melanoma disease stage or driver mutations, processed data was obtained from UCSC's Xena browser (https://tcga.xenahubs.net/), and visualized in R. Expression of INHBA in different cell types within the TME was examined using a publicly available single-cell RNA-seq data [10] (GSE72056). Processed data including the original cell type annotations was obtained from GEO and visualized in R.

Statistical analysis

Statistical tests were performed using the Prism software (GraphPad). Unless indicated, data represent the mean ± SEM of at least 2 independent experiments. When comparing two groups, normal distributions were analyzed by the Shapiro-Wilk normality test, and p-values calculated by Student's t-test (normal distribution) or Mann-Whitney's test (non-parametric test). One-way ANOVA was used to compare several groups of unpaired values. Kaplan-Meier survival curves were analyzed using the Gehan-Breslow-Wilcoxon test. Data points identified as outliers by the regression and outlier (ROUT) removal method in Prism 9 with a False Discovery Rate ≤1% were excluded. Power Analysis was waved by the animal experimentation authorities due to pre-existing data about the effect sizes of Activin-A induced tumor growth and cachexia. Tumor volumes at the endpoint were compared by ANOVA or Student's t-test, as indicated in the figure legends.

SUPPLEMENTARY REFERENCES

1. Falo LD, Kovacsovics-Bankowski M, Thompson K, Rock KL. Targeting antigen into the phagocytic pathway in vivo induces protective tumour immunity. Nat Med [Internet]. Nature Publishing Group; 1995 [cited 2021 Jun 4];1:649–53. Available from: http://www.nature.com/naturemedicine

2. Fuerer C, Nostro MC, Constam DB. Nodal⅐Gdf1 Heterodimers with Bound Prodomains Enable Serum-independent Nodal Signaling and Endoderm Differentiation. J Biol Chem [Internet]. in Press; 2014 [cited 2017 Jul 19];289:17854–71. Available from: https://www.ncbi.nlm.nih.gov/pmc/articles/PMC4067217/pdf/zbc17854.pdf

3. Donovan P, Dubey OA, Kallioinen S, Rogers KW, Muehlethaler K, Müller P, et al. Paracrine Activin-A signaling promotes melanoma growth and metastasis through immune evasion. J Invest Dermatol [Internet]. 2017;137:2578–87. Available from: https://www.ncbi.nlm.nih.gov/pubmed/28844941

4. Hampf M, Gossen M. A protocol for combined Photinus and Renilla luciferase quantification compatible with protein assays. Anal Biochem [Internet]. Academic Press Inc.; 2006 [cited 2020 Nov 9];356:94–9. Available from: https://pubmed.ncbi.nlm.nih.gov/16750160/

5. Neubert NJ, Schmittnaegel M, Bordry N, Nassiri S, Wald N, Martignier C, et al. T cell-induced CSF1 promotes melanoma resistance to PD1 blockade. Sci Transl Med [Internet]. 2018;10:eaan3311. Available from: http://stm.sciencemag.org/content/10/436/eaan3311.short

6. Feldman JP, Goldwasser R. A Mathematical Model for Tumor Volume Evaluation Using Two-Dimensions. Jpurnal Appl Quant methods. 2009;4:455–62.

7. Faget J, Groeneveld S, Boivin G, Sankar M, Zangger N, Garcia M, et al. Neutrophils and Snail Orchestrate the Establishment of a Pro-tumor Microenvironment in Lung Cancer. Cell Rep [Internet]. Elsevier B.V.; 2017 [cited 2021 Jun 14];21:3190–204. Available from: https://pubmed.ncbi.nlm.nih.gov/29241546/

8. Fuertes Marraco SA, Grosjean F, Duval A, Rosa M, Lavanchy C, Ashok D, et al. Novel murine dendritic cell lines: A powerful auxiliary tool for dendritic cell research. Front Immunol.

Frontiers; 2012;3:331.

9. Hugo W, Zaretsky JM, Sun L, Song C, Moreno BH, Hu-Lieskovan S, et al. Genomic and Transcriptomic Features of Response to Anti-PD-1 Therapy in Metastatic Melanoma. Cell [Internet]. Elsevier Inc.; 2016;165:35–44. Available from: http://dx.doi.org/10.1016/j.cell.2016.02.065

10. Tirosh I, Izar B, Prakadan SM, Wadsworth MH, Treacy D, Trombetta JJ, et al. Dissecting the multicellular ecosystem of metastatic melanoma by single-cell RNA-seq. Science (80-). 2016;352:189–96.

SUPPLEMENTARY TABLES

Supplementary Table 1. Activin-A induced changes in the B16-F1 TME

Population	Markers	Ctrl	βA	p-value
CD45+ leukocytes	CD45+	26.9% ± 7.6%	15.1% ± 8.7%	0.065
CD11b+ myeloid cells	CD11b+	51.4% ± 17.8%	70.8% ± 3.8%	0.0004
mMDSCs	CD11b+Ly6Chi	13.8% ± 6.5%	23.8% ± 6.7%	0.004
Macrophages	CD11b+F4/80+	12.7% ± 4.1%	17.3% ± 8.4%	0.17
Macrophage CD206 MFI	CD206	17142 ± 7961	25501 ± 5789	0.068
Neutrophils	CD11b+Ly6G+	4.5% ± 6.7%	4.3% ± 2.5%	0.24
Dendritic cells	CD11c+F4/80-	13.0% ± 6.3%	21.4% ± 4.4%	0.003
Dendritic cells MHC-II MFI	IA/IE	31396 ± 8305	30757 ± 4751	0.84
Dendritic cells CD80	CD80	1216 ± 3853	3853 ± 758	<0.0001
NK cells	NK1.1+CD3-	15.8% ± 12.1%	5.1% ± 2.9%	0.006
CD8 T-cells	CD3+CD8+	7.1% ± 4.0%	2.4% ± 1.0%	0.001
Tconv	CD3+CD4+FoxP3-	2.9% ± 1.4%	5.0% ± 2.2%	0.04
Tregs	CD3+CD4+FoxP3+	1.7% ± 0.8%	1.6% ± 1.6%	0.41
B-cells	CD19+B220+	1.1% ± 0.8%	3.4% ± 3.0%	0.07

Pinjusic K, *et al. J Immunother Cancer* 2022; 10:e004533. doi: 10.1136/jitc-2022-004533

Supplementary Table 2. qPCR primers

Primer Name	Sequence	Species
Gapdh fw	ACTGAGGACCAGGTTGTCTCC	Mus musculus
Gapdh rv	GTTGGGATAGGGCCTCTCTTGC	Mus musculus
Acvr1b fw	GGGTGGGGACCAAACGATAC	Mus musculus
Acvr1b rv	TCGGAGGGCACTAAGTCGTA	Mus musculus
Acvr1c fw	AGACGGTGATGCTGAGACACGA	Mus musculus
Acvr1c rv	GACCATTCCAGCCACAGTCACT	Mus musculus
Acvr2b fw	ATTGCTACGACAGGCAGGAG	Mus musculus
Acvr2b rv	GTGGCTCGTACGTGACTTCT	Mus musculus
Acvr2a fw	AAGTTCGAGGCTGGCAAGTCTG	Mus musculus
Acvr2a rv	CCTCAGAAATGCGTCCCTTTGG	Mus musculus
IFNy fw	TGAACGCTACACACTGCATCTTGG	Mus musculus
IFNy rv	CGACTCCTTTTCCGCTTCCTGAG	Mus musculus
IL10 fw	GCTCTTACTGACTGGCATGAG	Mus musculus
IL10 rv	CGCAGCTCTAGGAGCATGTG	Mus musculus
IL1b fw	TGCCACCTTTTGACAGTGATGAGA	Mus musculus
IL1b rv	TCATCAGGACAGCCCAGGTCA	Mus musculus
IL6 fw	ACCACGGCCTTCCCTACTTC	Mus musculus
IL6 rv	TTGCCATTGCACAACTCTTTTCTCA	Mus musculus
TNFa fw	CCCACGTCGTAGCAAACCA	Mus musculus
TNFa rv	ACAAGGTACAACCCATCGGC	Mus musculus
IL2 fw	ACTAAAGGGCTCTGACAACAC	Mus musculus
IL2 rv	CCTCAGAAAGTCCACCACAGT	Mus musculus
Perforin fw	ACACAGTAGAGTGTCGCATGTAC	Mus musculus
Perforin rv	GTGGAGCTGTTAAAGTTGCGGG	Mus musculus
Granzyme B fw	CAGGAGAAGACCCAGCAAGTCA	Mus musculus
Granzyme B rv	CTCACAGCTCTAGTCCTCTTGG	Mus musculus
Inhba fw	CCTCTGGCTATCACGCCAAT	Mus musculus
Inhba rv	ACATGGGTCTCAGCTTGGTG	Mus musculus
Fst fw	AGTAAGTCGGATGAGCCGGT	Mus musculus
Fst rv	TTCACTTCAAGAAGCACGCC	Mus musculus

Supplementary Table 3. Antibodies used for immune-profiling

Reagent	Source	Clone	Reference
CD103 PE	eBioscience	2.00E+07	12-1031-82
CD11b BV711	BioLegend	M1/70	101241
CD11c BV421	BioLegend	N418	117330
CD172a (SIRPα) PerCP/Cy5.5	eBioscience	P84	144009
CD19 BV510	BioLegend	6D5	115545
CD197 (CCR7) AlexaFluor488	eBioscience	4B12	120112
CD206 FITC	BioLegend	C068C2	141703
CD25 APCeFluor780	eBioscience	PC61.5	47-0251-82
CD25 PE	BioLegend	PC61	102007
CD3 APC-eFluor780	eBioscience	500A2	47-0033-82
CD3 PE	BioLegend	145-2C11	100308
CD3e PE-Cy5.5	eBioscience	145-2C11	35-0031-82
CD4 BV785	BioLegend	RM4-5	100552
CD4 PE-Cy7	eBioscience	GK1.5	25-0041-82
CD45 APC	eBioscience	30-F11	17-0451-82
CD45 PerCP	BioLegend	30-F11	103129
CD45.1 BV421	BioLegend	A20	110731
CD45.2 BUV737	BD Horizon	104	564880
CD45R (B220) APC	Miltenyi	RA3-6B2	130-102-259
CD69 BV711	BioLegend	H1.2F3	104537
CD8 BV510	BD Horizon	53-6.7	563068
CD80	Miltenyi	16-10A1	130-102-372
CXCL9 (MIG) AlexaFluor647	eBioscience	MIG-2F5.5	515606
F4/80 BV605	BioLegend	BM8	123133
FoxP3 PE-eFluor610	eBioscience	FJK-16s	61-5773-82
GranzymeB AlexaFluor647	BioLegend	GB11	515406
H2Kb FITC	BioLegend	AF6-88.5	116505
H2Kb-SIINFEKL APC	BioLegend	25-D1.16	141605
IA/IE (MHC-II) AlexaFluor700	BioLegend	M5/114.15.2	107622
IA/IE (MHC-II) APC-Cy7	BioLegend	M5/114.15.2	107627
IFNγ PE	BioLegend	XMG1.2	505808
Ki67 eFluor450	Invitrogen	SolA15	48-5698-80
Ly6C AlexaFluor700	BioLegend	HK1.4	128023
Ly6C PerCP/Cy5.5	BioLegend	HK1.4	128012
Ly6G FITC	BioLegend	1A8	127605
Ly6G PE	BioLegend	1A8	127607
NK1.1 BV650	BioLegend	PK136	108735
PD1 PE/Cy7	BioLegend	RMP1-30	109109
TNFα PE-Cy7	BioLegend	MP6-XT22	506323
XCR1 APC/Cy7	eBioscience	ZET	148223

Pinjusic K, *et al. J Immunother Cancer* 2022; 10:e004533. doi: 10.1136/jitc-2022-004533

SUPPLEMENTARY FIGURES

Supplementary Figure 1. Effects of *INHBA* on the composition, cell proliferation, and gene expression of immune infiltrates in B16-F1 melanoma.

A, Circulating Activin-A plasma concentrations in B16F1-Ctrl or -βA tumor-bearing mice quantified by ELISA. Error bars, SEM (n = 5 per group); **p<0.01, Student's t-test. **B-H,** qPCR analyses of (B) *Gzmb*, (C) *Perf1*, (D) *Il1b*, (E) *Il2*, (F) *Il10*, (G) *Ifng*, and (H) *Tnfa*, in whole tumor extracts normalized to *Gapdh* expression (n = 6-7 Ctrl, and 10 βA tumors). **I,** Percentage of B cells ($B220^+CD19^+$) in $CD45^+$ cells in tumors (n = 4 Ctrl, 7 βA tumors). **J-M,** Quantification of $Ki67^+$ proliferating cells in dissociated B16F1-Ctrl or -βA tumors stained by flow cytometry for (J) $CD3^+CD8^+$, (K) $CD3^-NK1.1^+$, (L) $CD4^+FoxP3^+$, (M) $CD4^+FoxP3^-$, Error bars, SEM (n = 7-9); *p<0.05, **p<0.01, ***p<0.001, ****p<0.0001, Student's t-test. **N,** Summary of *INHBA*-induced changes observed in the TME of B16-F1 melanoma.

Supplementary Figure 2. Antigen cross-presentation in blood and lymphoid organs and analysis of CD4/CD8 cell depletion in B16.OVA-βA melanoma-bearing mice.

A, H2Kb expression (left) and H2Kb-SIINFEKL presentation (right) in B16.OVA-Ctrl and -βA stable cell lines treated with or without 20 µg/mL IFNγ for 24 hrs. Western blot of OVA and γ-tubulin expression in B16.OVA-Ctrl and -βA stable cell lines. **B,** As in (A), but for B16F1-Ctrl.OVA and –βA.OVA cell lines. **C-F,** Flow cytometric analysis of the frequency of (C) $CD11c^+$ and (D) $CD11c^+CD8^+$ APCs in $CD45^+$, and (E) H2Kb-SIINFEKL presentation by $CD11c^+$ APCs, and (F) H2Kb expression in $CD11c^+$ APCs in dLNs of B16.OVA-Ctrl and B16.OVA-βA tumor-bearing mice (n = 4). **G-H,** Flow cytometry of (G) $CD4^+$ and $CD8^+$ T cells in blood and tumors of B16.OVA-Ctrl and -βA tumor-bearing mice, and (H) quantification of $CD3^+CD8^+$ T cells at the endpoint after injection of the indicated antibodies. Error bars, SEM (n = 4-5); *p<0.05, **p<0.01, ***p<0.001, ****p<0.0001, ordinary one-way ANOVA with Holm-Šídák correction for multiple comparisons.

Pinjusic K, *et al. J Immunother Cancer* 2022; 10:e004533. doi: 10.1136/jitc-2022-004533

Supplementary Figure 3. Analysis of *in vitro* activated T cells and their effect on tumor immune infiltrates after adoptive transfer in B16-F1 melanoma-bearing mice.

A, RT-qPCR analysis of *Acvr2a*, *Acvr2b*, *Alk4,* and *Alk7* mRNA expression in CD8-depleted splenocytes of naïve mice (-) and in *in vitro* differentiated $CD8^+$ T cells (+), normalized to *Gapdh* mRNA expression. Error bars, SEM (n = 3 biological replicates); p-values, Student's t-test. **B,** Representative Western blots of pSmad2, total Smad2 and γ-tubulin (loading control) in naïve $CD8^+$ T cells and CD8 negative splenocytes isolated from OT-I mice treated for 2 hrs with 20 ng/ml TGFβ1 or Activin-A (n = 3 experiments). **C,** Western blots of pSmad2, total Smad2, and Gapdh (loading control) in OT-I T cells stimulated during 4 days with 20 ng/ml Activin-A (ActA) or without (CTRL), together with OVA peptide (SIINFEKL) and IL2. Extracts of B16F1 cells treated without (-) or with doxycycline (Dox) to induce constitutively active mutant Alk4 (caALK4) were used as controls [3]. **D,** Representative Western blots of pSmad2 and γ-tubulin (loading control) in OT-I T cells treated for 2h with 20 ng/ml TGFβ or Activin-A after 4 days of *in vitro* activation (n = 3 independent experiments). **E,** Mean fluorescence intensities of CD25, CD69, and PD1 (n = 4 experiments) and TNFα, IFNγ, Granzyme B stainings in OT-I T cells (n = 3 experiments) that were activated during 4 days with or without Activin-A. Error bars, SEM; p-values, 2way ANOVA with Tukey's correction for multiple comparison. **F**, Comparison of B16.OVA and B16F1.OVA-Ctrl versus –βA tumor cell killing, and non-OVA B16 cell killing in 1:1 co-culture with activated OT-I T cells (n = 4-5 independent experiments). Error bars, SEM, p-values, ****p<0.0001, ordinary one-way ANOVA with Holm-Šídák correction for multiple comparisons **G-J,** Quantification of (G) $CD45^+$, (H) $CD11b^+$, (I) $CD4^+$ T cells, and (J) endogenous $CD45.2^+CD8^+$ T cells per mg of B16F1-Ctrl.OVA or B16F1-βA.OVA tumor one week after the injection of activated OT-I T cells or PBS control. Error bars, SEM (n = 7-8), *p<0.05, **p<0.01, ***p<0.001, ****p<0.0001, ordinary one-way ANOVA with Holm-Šídák correction for multiple comparisons. **K-M,** Mean fluorescence intensity of (K) IFNγ, (L) Granzyme B, and (M) TNFα protein staining in OT-I T cells analyzed by flow cytometry one week after the transfer into B16F1-Ctrl.OVA or -βA.OVA tumor-bearing mice. Error bars, SEM (n=7-8); p<0.05, **p<0.01, ***p<0.001, ****p<0.0001 (Student's t-test).

Supplementary Figure 4. Characterization of YUMM3.3 and iBIP2 2891L cell lines.

A, RT-qPCR analyses of endogenous *Inhba* expression in B16F1 (n = 6), YUMM3.3 (n = 5), and iBIP2 2891L cells (n = 6) relative to *Gapdh* expression. **B,** Anti-Activin-A Western blot of B16F1, YUMM3.3, and iBIP2 2891L cell-conditioned media on non-reducing gels. Predicted molecular weights of myc-tagged Activin-A precursor (proβA_2), hemicleaved form (proβA:βA), and mature form (βA_2) are indicated. Asterisk marks endogenous mature Activin-A. UT: untransfected; Ctrl: Empty control lentivirus; βA: *myc-INHBA* lentivirus. **C,** RT-qPCR analyses of endogenous *Fst* expression in B16F1, YUMM3.3, and iBIP2 2891L cell lines relative to *Gapdh* expression (n = 3). **D,** CAGA-Luc induction in HepG2 reporter cells after overnight incubation with 0, 20, or 40 ng/ml Activin-A in the control medium or in the SN collected from YUMM3.3 cells (n = 2). **E,** CAGA-Luc induction after overnight incubation with iBIP2 2891L supernatant alone or in presence of an increasing concentration of YUMM3.3 supernatants. Error bars, SEM (n = 3). **F,** Alamar blue cell viability staining in YUMM3.3-Ctrl and -βA cell cultures, and in YUMM3.3 cells treated with 20 ng/ml Activin-A (top, n = 3-4), or in cultured iBIP2 2891L Mock-Fc and AIIB-Fc cells (below, n = 2); Error bars, SEM, *p<0.05, **p<0.01, ***p<0.001, ****p<0.0001, Student’s t-test.

Pinjusic K, *et al. J Immunother Cancer* 2022; 10:e004533. doi: 10.1136/jitc-2022-004533

Supplementary Figure 5. Activin-A accelerates melanoma growth independently of CSF1R$^+$ TAMs.

A-D, Frequency of (A) CD11b$^+$, (B) CD11b$^+$Ly6C^{hi}, (C) CD11b$^+$F4/80^{hi}, and (D) CD11b$^+$F4/80$^-$ cells in CD45$^+$ immune infiltrates from YUMM3.3-Ctrl versus –βA tumors (left), or from iBIP2 Mock-Fc versus AIIB-Fc tumors (right) analysed by flow cytometry at endpoints. **E,** Growth curves of YUMM3.3-Ctrl and -βA tumors treated with αCSF1R or control IgG antibodies. Error bars, SEM (n=4-5).; *p<0.05, **p<0.01, ***p<0.001, ****p<0.0001, Student's t-test. **F,** The ratio of CD8/CD4 cells in the tumors shown in (E) at the endpoint. Error bars, SEM (n = 4-5); *p<0.05, **p<0.01, ***p<0.001, ****p<0.0001, 2-way ANOVA with Tukey's correction for multiple comparison. **G,** Representative flow cytometry gating panels (left), and quantification of F4/80^{hi} TAM depletion in YUMM3.3-Ctrl and -βA tumors (right) after αCSF1R or control IgG administration. **H-K,** Expression of (H) IFNγ and (I) TNFα in CD8$^+$ T, and of (J) IFNγ and (K) TNFα in CD4$^+$ T cells at the endpoint. Error bars, SEM (n = 4-5); *p<0.05, **p<0.01, ***p<0.001, ****p<0.0001, 2-way ANOVA with Tukey's correction for multiple comparison.

Supplementary Figure 6. Activin-A directly impairs CXCL9/10 production by immortalized cDC1 cells

A, Illustration of the experimental procedure. Immortalized mouse cDC1 cells [8] were activated with 5 µg/ml LPS + 10 ng/ml IFNγ and, where indicated, with 50 ng/ml ActA, TGFβ, BMP4, or 10 µM SB inhibitor during 24 hrs. Production of CXCL9 was analyzed by intracellular cytokine staining and flow cytometry after incubation with Golgi plug for 3 hrs. Secreted chemokines in the SN were quantified by ELISA. **B,** Histogram of one representative immunofluorescent staining (left panel), and quantification of the mean fluorescence intensity (MFI) of intracellular CXCL9 (right panel) in LPS-activated cDC1 cells that were treated as indicated (n = 3-5). **C, D,** Secretion of (C) CXCL9 and (D) CXCL10 in the SN of cDC1 cultures measured by ELISA (n = 3-4). **E,** Expression (MFI) of the cell proliferation marker Ki67 in cDC1 cells that were treated as indicated. Error bars, SEM (n = 2-4); *p<0.05, **p<0.01, ***p<0.001, ****p<0.0001, ordinary one-way ANOVA with Holm-Šídák correction for multiple comparisons. All MFI values were normalized to those of cells activated with LPS alone (control).

Pinjusic K, *et al. J Immunother Cancer* 2022; 10:e004533. doi: 10.1136/jitc-2022-004533

Supplementary Figure 7. Expression of immune regulators that were not modulated by melanoma-derived Activin-A.

A-P, Quantification of (A) CCL2, (B) CCL5, (C) CXCL5, (D) CCL3, (E) CCL4, (F) CCL20, (G) IL5, (H) IL2, (I) IL6, (J) TNFα, (K) IL9, (L) IL21, (M) IL13, (N) IL22, (O) IL17A, and (P) IL17F secreted from *ex vivo* cultured dissociated YUMM3.3-Ctrl (n = 5-6) and -βA tumors (n = 8). Error bars, SEM; *p < 0.05, **p < 0.01, ***p < 0.001, and ****p < 0.0001, Mann-Whitney test for non-parametric data and a two-tailed t-test for parametric data. **Q,** Relative *Tgfb1* mRNA levels in total YUMM3.3-Ctrl and –βA tumor extracts measured by RT-qPCR and normalized to *Gapdh*. Error bars, SEM (n = 4); p-value, Student's t-test. **R,** Growth curves of YUMM3.3-Ctrl and -βA tumors treated with αIL-4 or IgG and tumor volumes measured at the endpoint. Error bars, SEM (n = 4-5); *p < 0.05, **p < 0.01, ***p < 0.001, and ****p < 0.0001, ordinary one-way ANOVA with Holm-Šídák correction for multiple comparisons. **S,** Representative histograms showing CXCL9 expression in MoMac and DC subsets in YUMM3.3-Ctrl and –βA tumors.

Supplementary Figure 8. Correlation of *INHBA* expression with the survival of melanoma patients.

A, Survival of patients with *INHBA* low versus high expression in TCGA data plotted using TIMER (http://timer.cistrome.org/). **B,** Correlation of *INHBA* expression with melanoma disease stage. **C,** Kaplan-Meier survival analysis of YUMM3.3-Ctrl and –βA tumor-bearing mice treated with ICB therapy. Mice were considered dead when the tumor reached volume >1 cm^3 (n = 11-12 per group). Curves were compared using the Log-rank (Mantel-Cox) test.

Supplementary Figure 9. Gating strategy for a 16-color flow cytometry antibody panel used for TILs profiling

Supplementary Figure 10. Gating strategy used in Fig. 2 and S2

A B, Analyses of (A) OVA-specific T cells in tumors and (B) SIINFEKL presentation by APCs in tumors and LNs.

Supplementary Figure 11. Gating strategy used for ATCT analyses in Fig. 3 and S3

A B, Analyses of OT-I cells after the ATCT in (A) tumors and (B) LNs

Supplementary Figure 12. Gating strategy used in Fig. 5 and S7 to analyze CXCL9 expression in tumors

Supplementary Figure 1

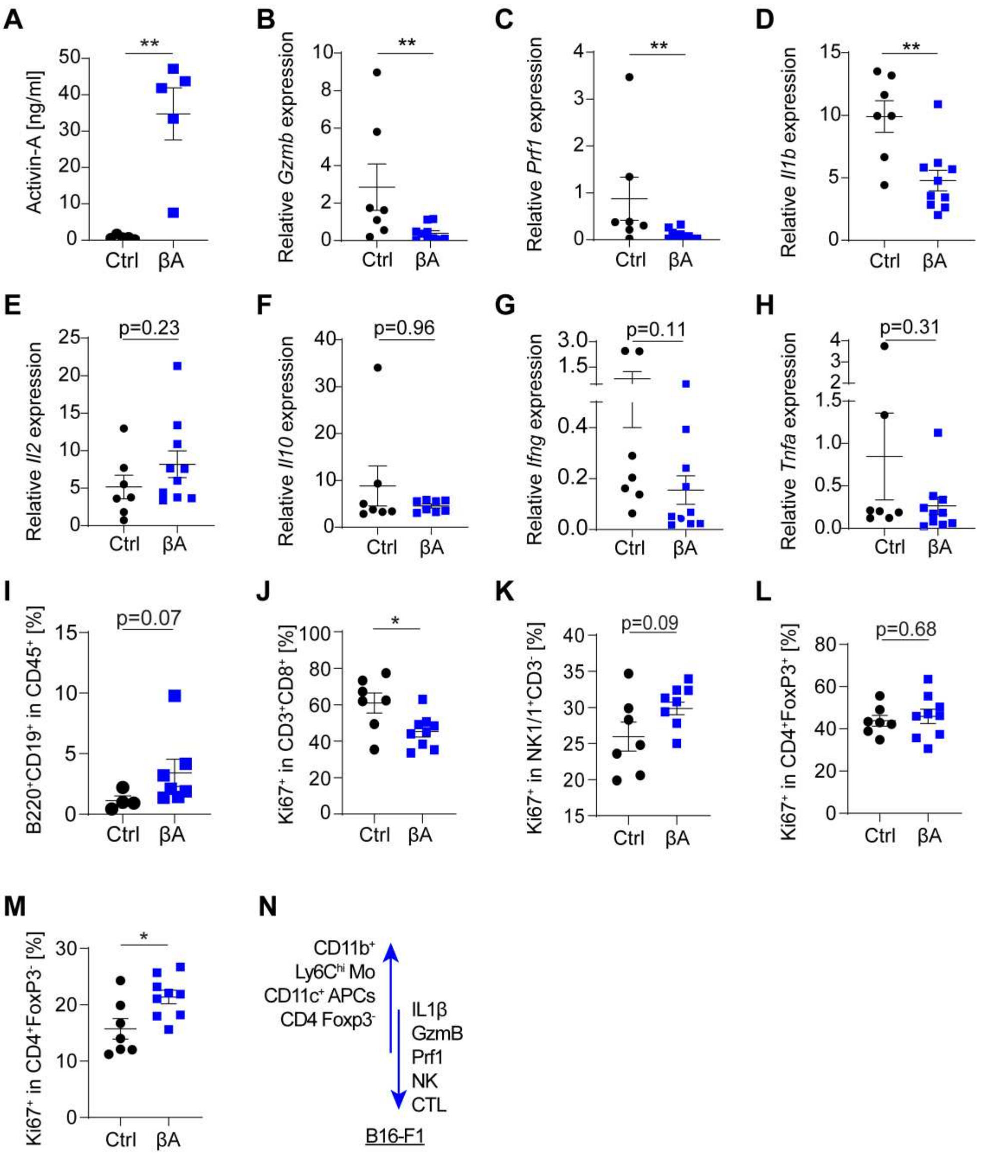

Pinjusic K, *et al. J Immunother Cancer* 2022; 10:e004533. doi: 10.1136/jitc-2022-004533

Supplementary Figure 2

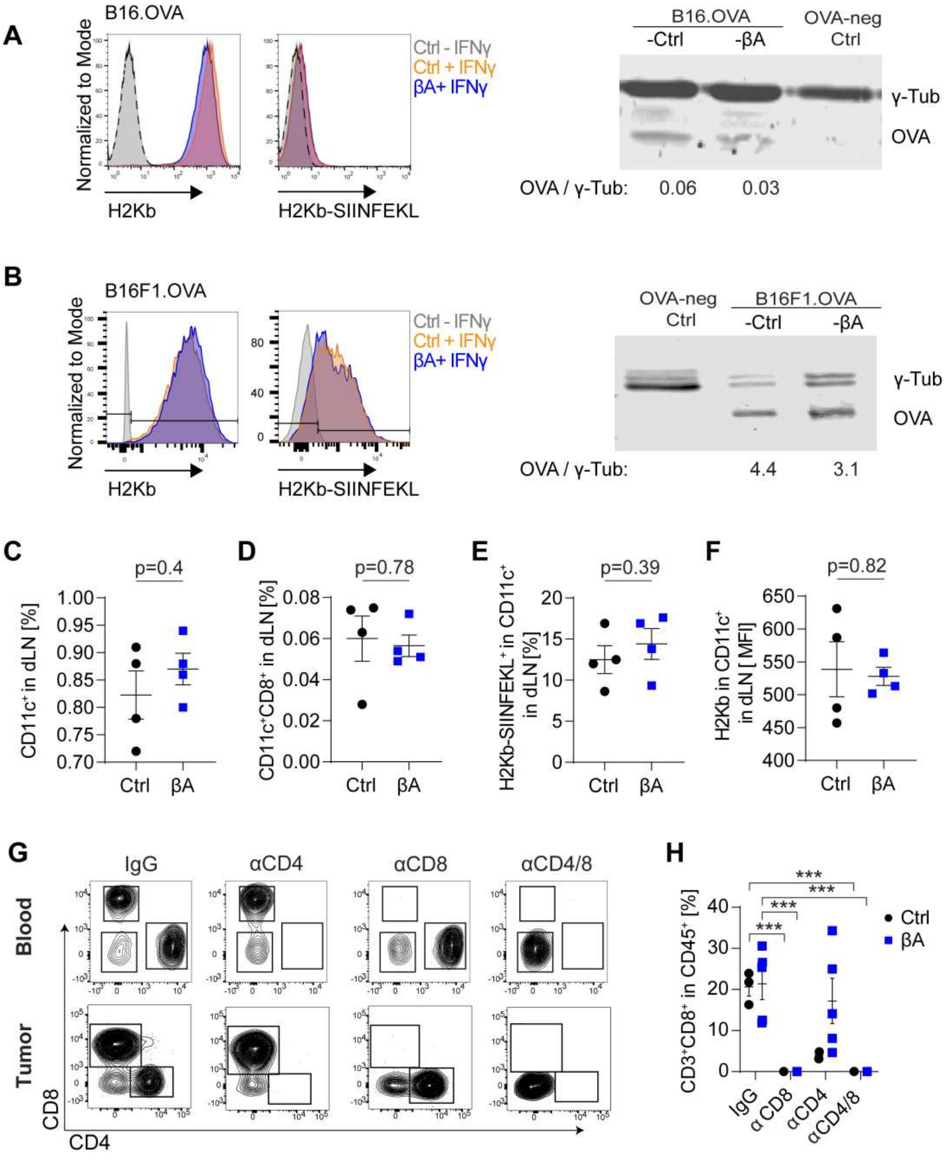

Supplementary Figure 3

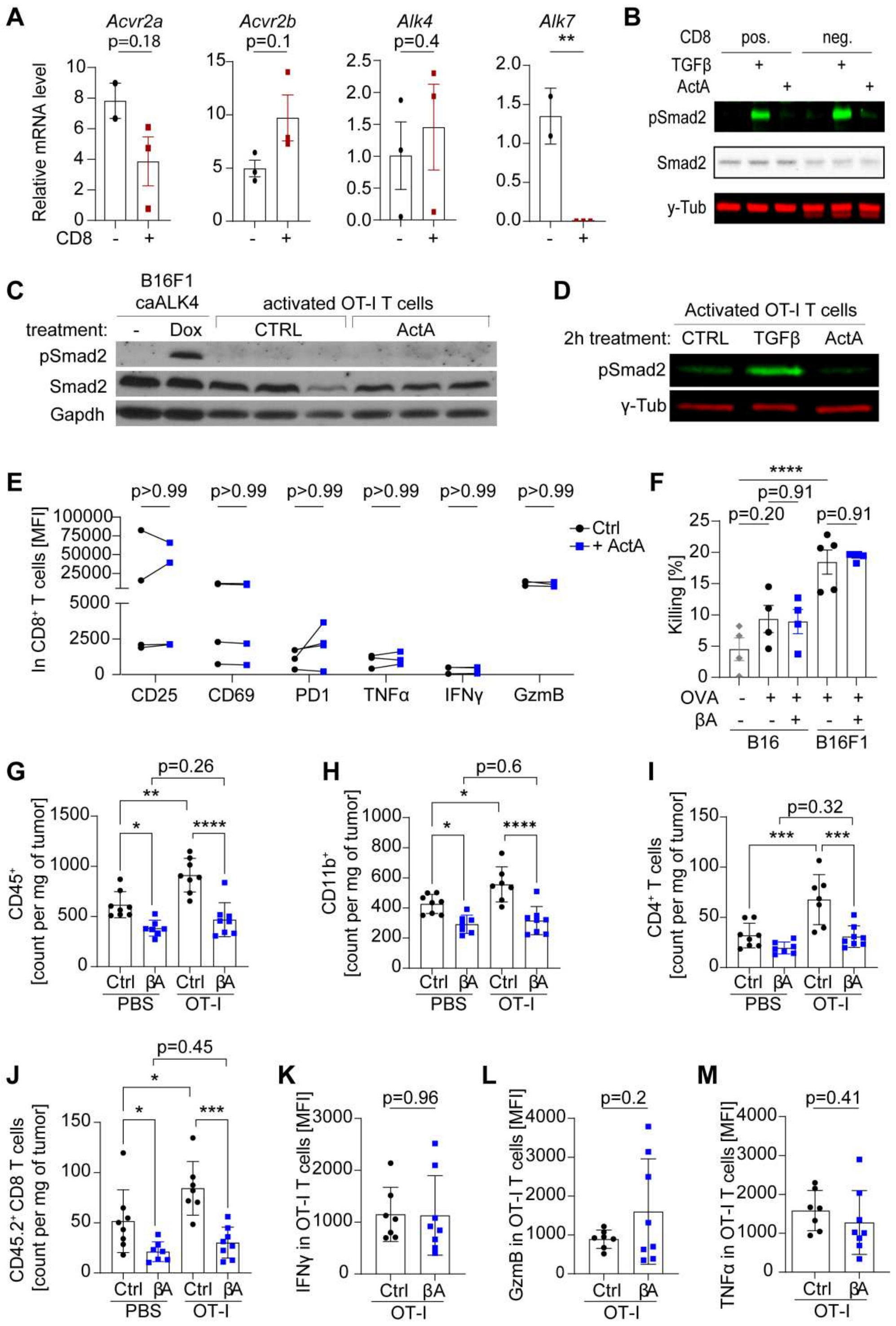

Pinjusic K, et al. J Immunother Cancer 2022; 10:e004533. doi: 10.1136/jitc-2022-004533

Supplementary Figure 4

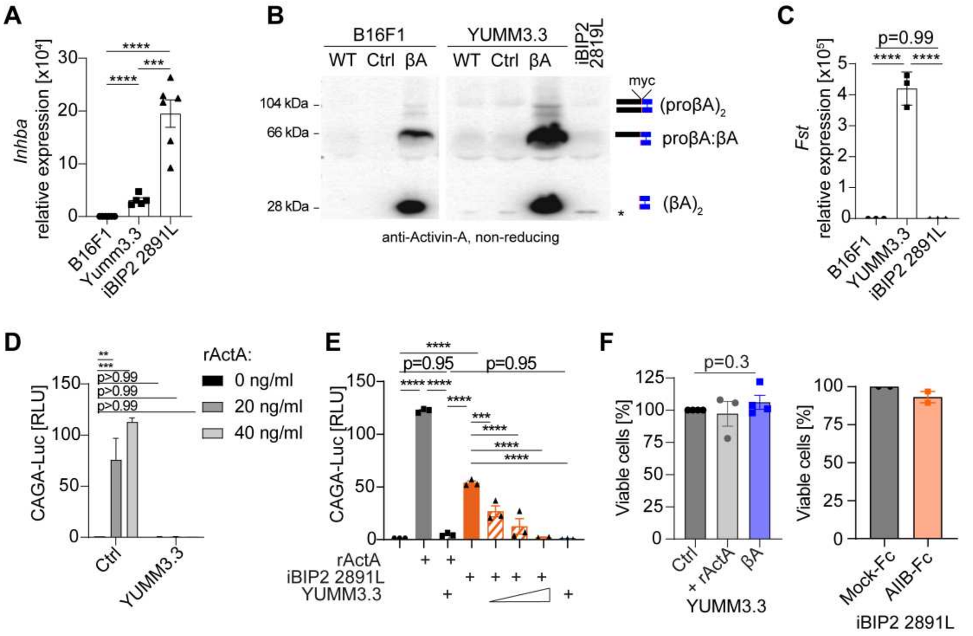

Supplementary Figure 5

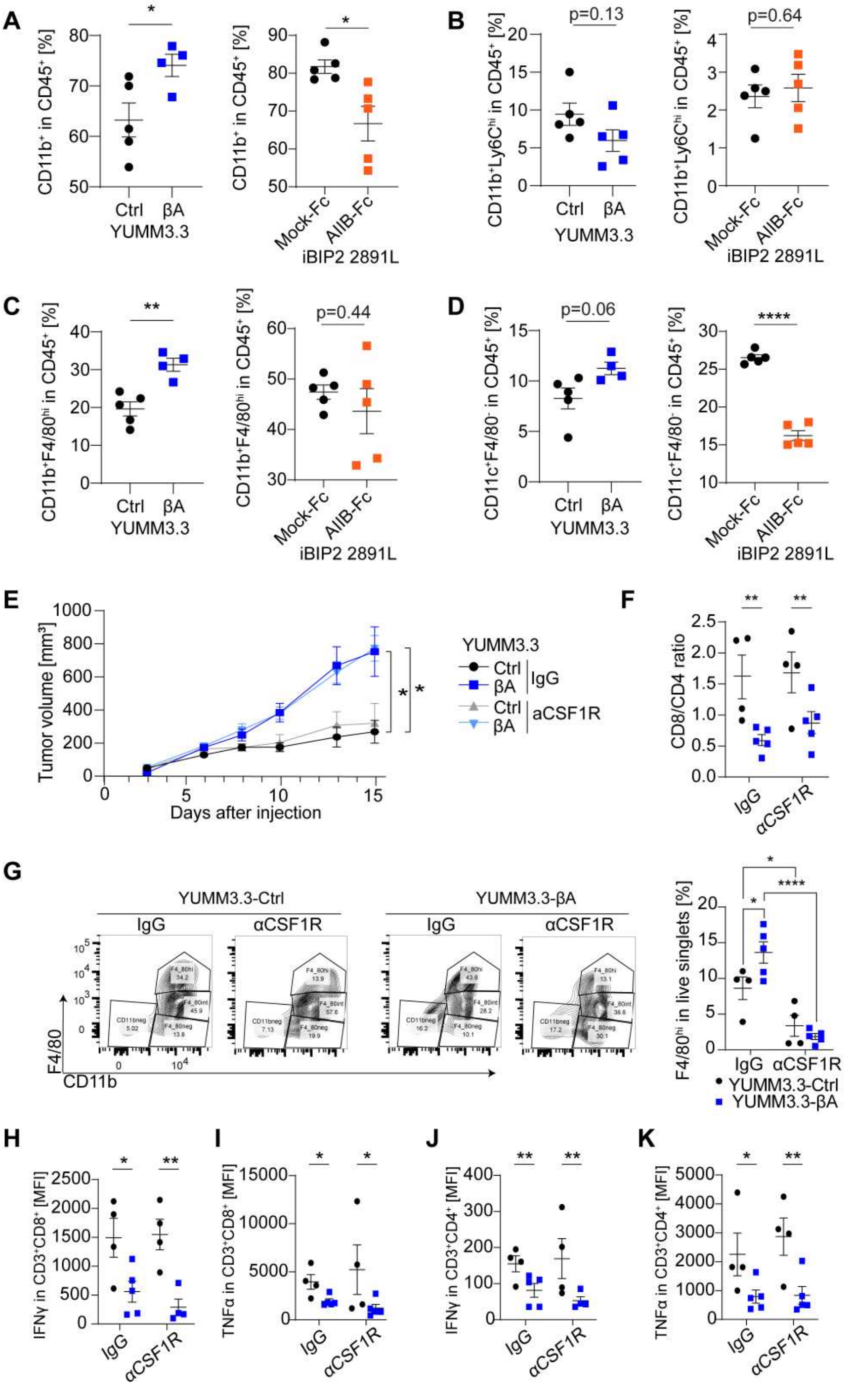

Pinjusic K, *et al. J Immunother Cancer* 2022; 10:e004533. doi: 10.1136/jitc-2022-004533

Supplementary Figure 6

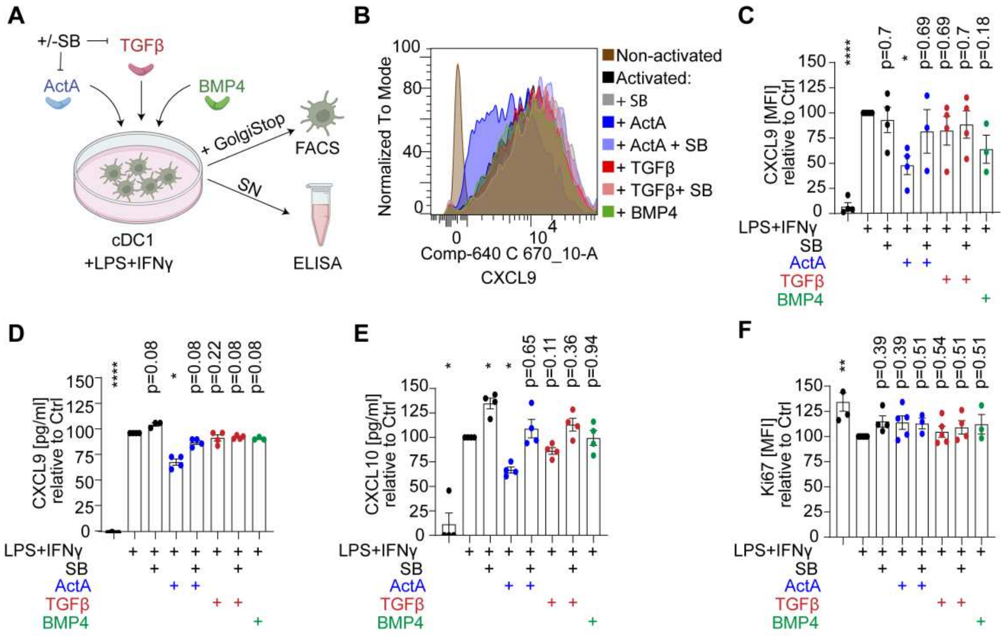

Pinjusic K, et al. J Immunother Cancer 2022; 10:e004533. doi: 10.1136/jitc-2022-004533

Supplementary Figure 7

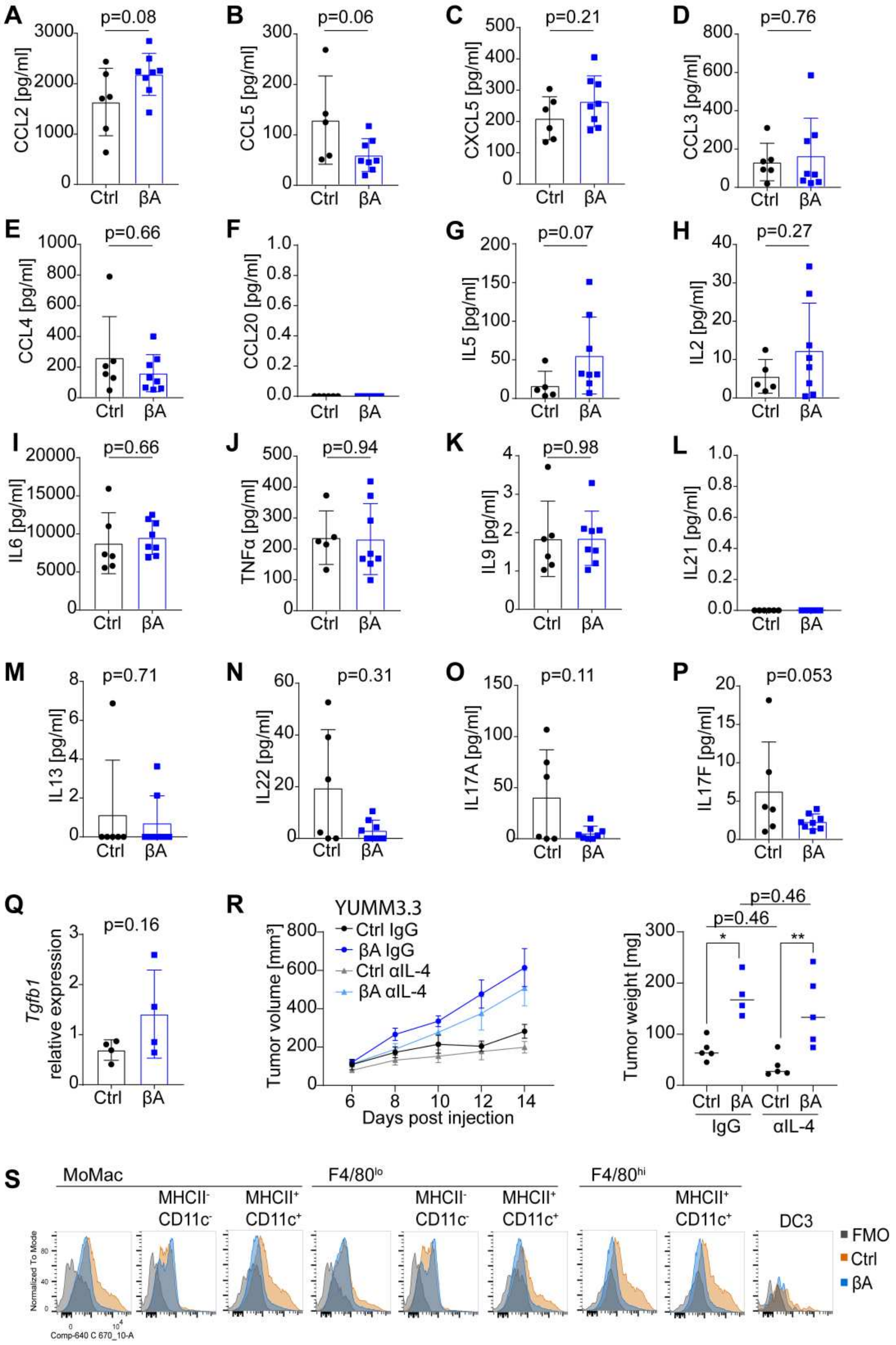

Pinjusic K, *et al. J Immunother Cancer* 2022; 10:e004533. doi: 10.1136/jitc-2022-004533

Supplementary Figure 8

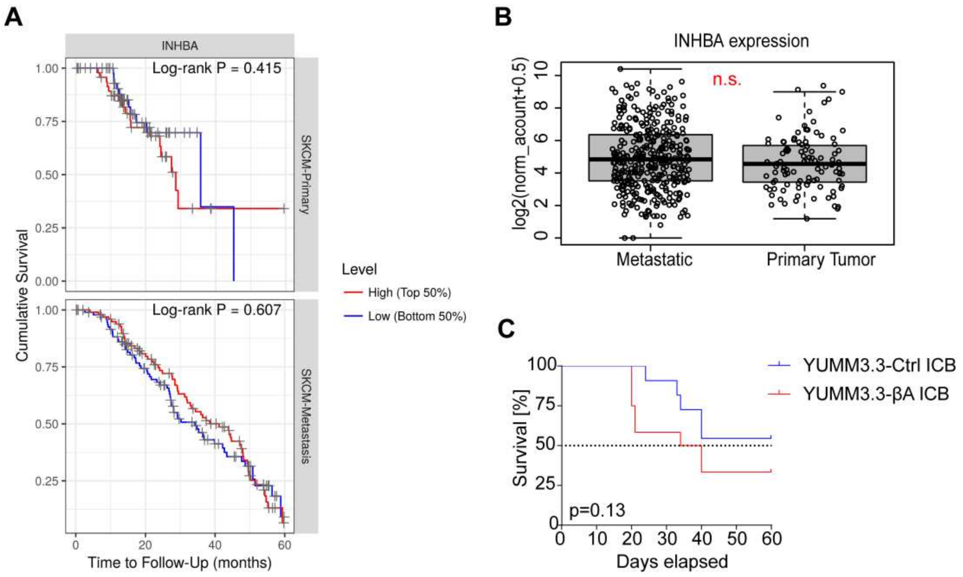

Pinjusic K, et al. J Immunother Cancer 2022; 10:e004533. doi: 10.1136/jitc-2022-004533

Supplementary Figure 9

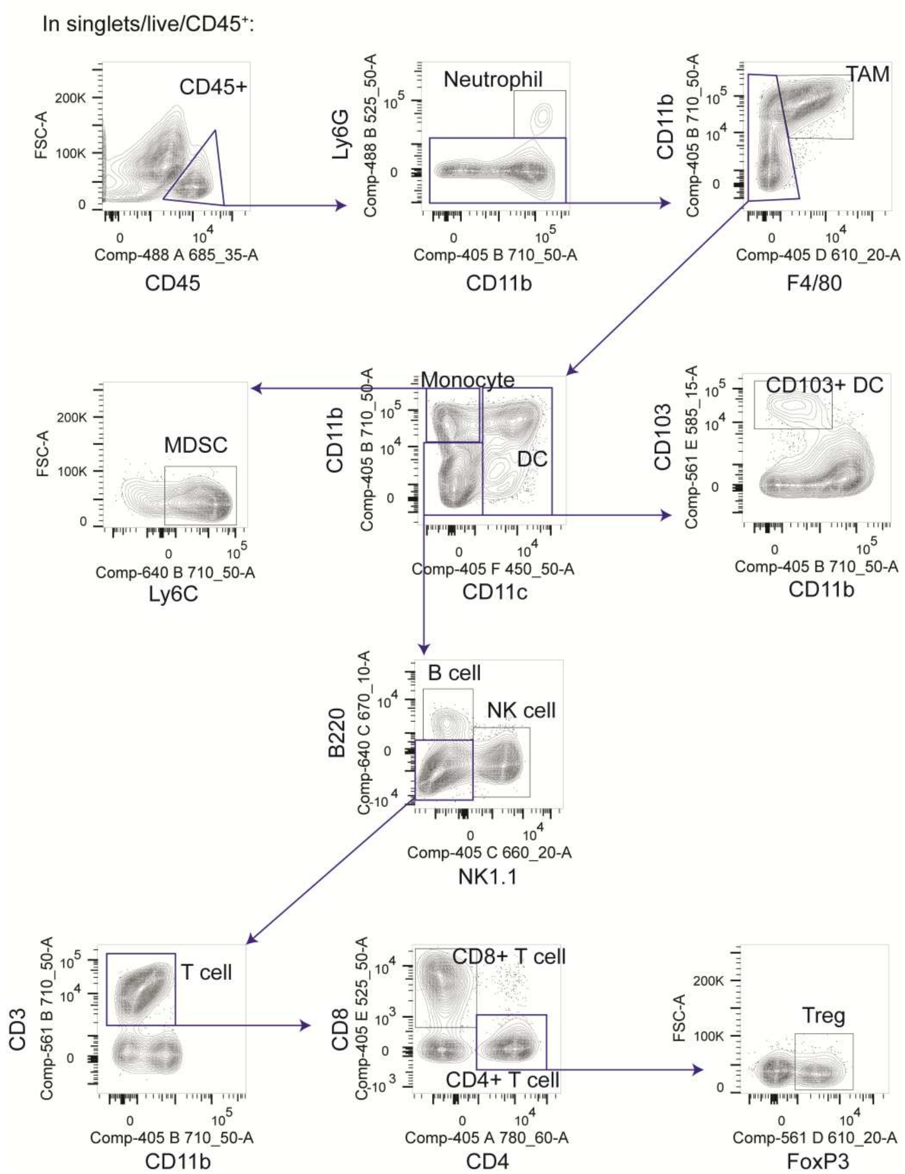

Supplementary Figure 10

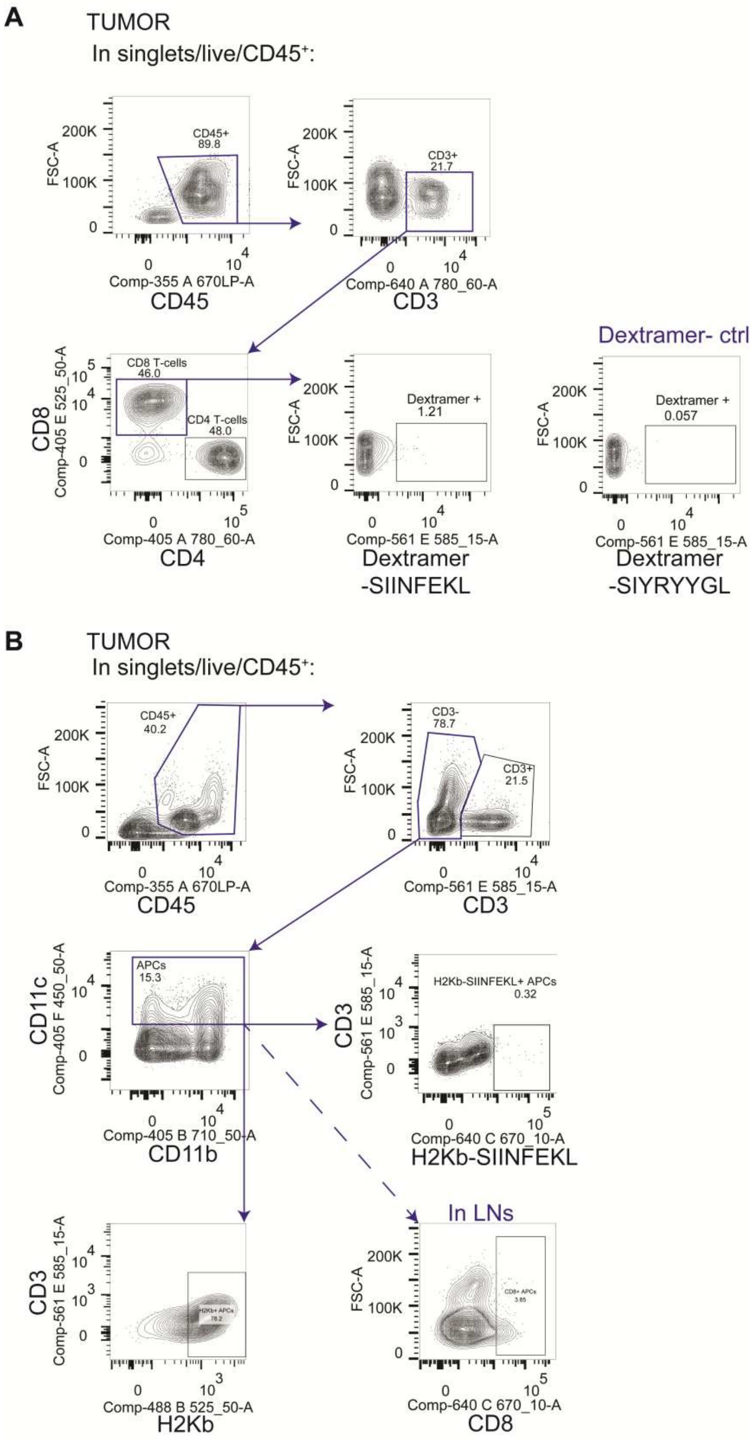

Pinjusic K, *et al. J Immunother Cancer* 2022; 10:e004533. doi: 10.1136/jitc-2022-004533

Supplementary Figure 11

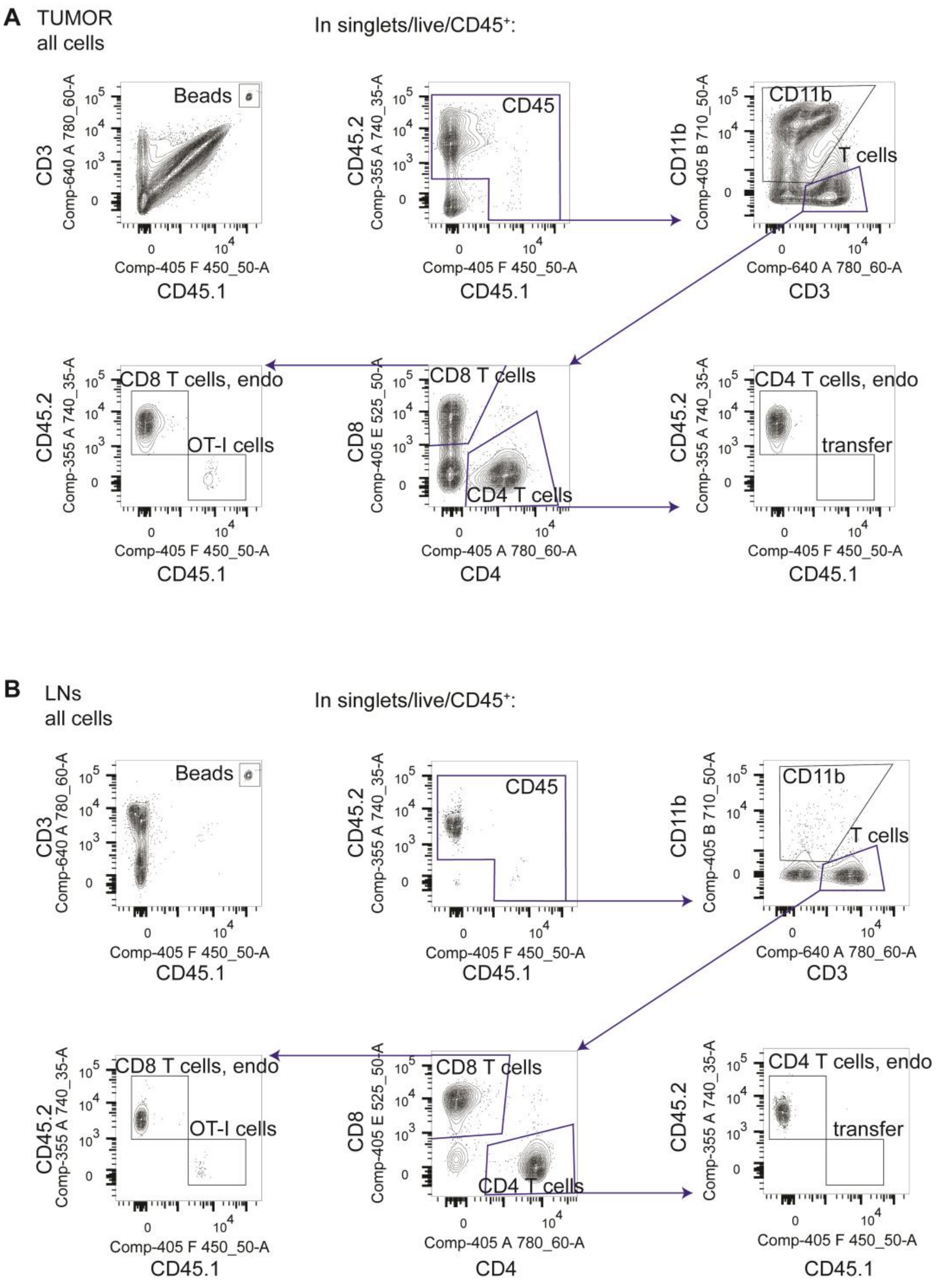

Supplementary Figure 12

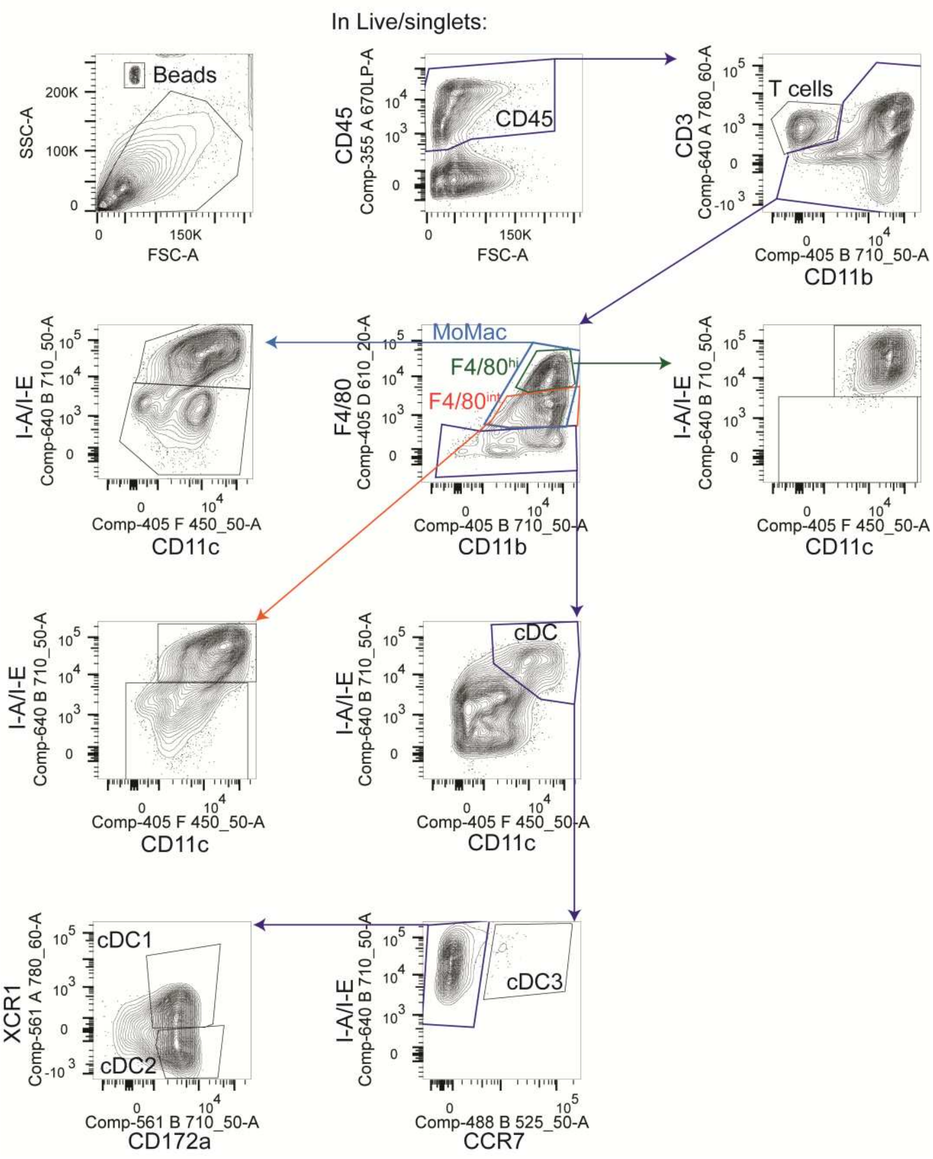

Pinjusic K, *et al. J Immunother Cancer* 2022; 10:e004533. doi: 10.1136/jitc-2022-004533
