# Supplementary material for: Activin-A impairs CD8 T cell-mediated immunity and immune checkpoint therapy response in melanoma
Source: J Immunother Cancer. 2022 May 16;10(5):e004533. doi: 10.1136/jitc-2022-004533 (PMC9125758; doi:10.1136/jitc-2022-004533)
Supplement: Supplementary data [file jitc-2022-004533supp002.pdf]

Activin-A impairs CD8 T cell-mediated immunity and immune checkpoint therapy response in melanoma

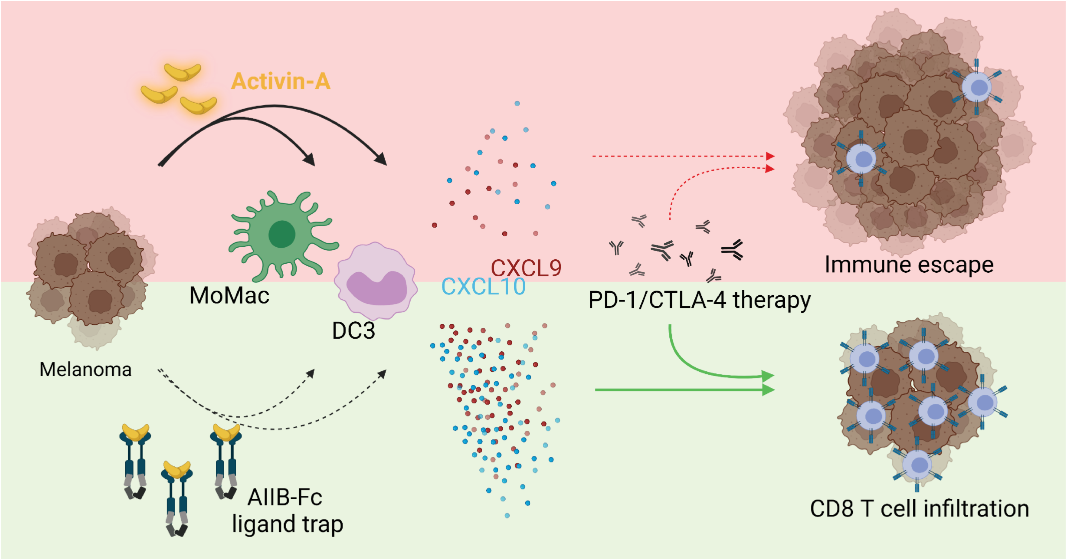

Authors
Katarina Pinjusic, Olivier A. Dubey, Olga Egorova, Sina Nassiri, Etienne Meylan, Julien Faget, and Daniel B. Constam

Correspondence
daniel.constam@epfl.ch

In Brief
Activin-A promotes immunotherapy resistance and tumor immune evasion by indirectly inhibiting CTL accumulation and function. Thus, Activin-A inhibition holds promise to boost anti-tumor immunity and immunotherapy.
